# Bioprospecting of Soil-Derived Actinobacteria Along the Alar-Hotan Desert Highway in the Taklamakan Desert

**DOI:** 10.3389/fmicb.2021.604999

**Published:** 2021-03-15

**Authors:** Shaowei Liu, Ting Wang, Qinpei Lu, Feina Li, Gang Wu, Zhongke Jiang, Xugela Habden, Lin Liu, Xiaolin Zhang, Dmitry A. Lukianov, Ilya A. Osterman, Petr V. Sergiev, Olga A. Dontsova, Chenghang Sun

**Affiliations:** ^1^Department of Microbial Chemistry, Institute of Medicinal Biotechnology, Chinese Academy of Medical Sciences & Peking Union Medical College, Beijing, China; ^2^Beijing Key Laboratory of Antimicrobial Agents, Institute of Medicinal Biotechnology, Chinese Academy of Medical Sciences & Peking Union Medical College, Beijing, China; ^3^College of Life Science, Xinjiang Normal University, Urumchi, China; ^4^School of Traditional Chinese Pharmacy, China Pharmaceutical University, Nanjing, China; ^5^School of Life Science and Technology, China Pharmaceutical University, Nanjing, China; ^6^Center of Life Sciences, Skolkovo Institute of Science and Technology, Moscow, Russia; ^7^Department of Chemistry, A.N. Belozersky Institute of Physico-Chemical Biology, Lomonosov Moscow State University, Moscow, Russia; ^8^Shemyakin-Ovchinnikov Institute of Bioorganic Chemistry, Russian Academy of Sciences, Moscow, Russia

**Keywords:** actinobacteria, diversity, macrolides, Taklamakan desert, novel species, antibacterial metabolites

## Abstract

Taklamakan desert is known as the largest dunefield in China and as the second largest shifting sand desert in the world. Although with long history and glorious culture, the Taklamakan desert remains largely unexplored and numerous microorganisms have not been harvested in culture or taxonomically identified yet. The main objective of this study is to explore the diversity, novelty, and pharmacological potential of the cultivable actinomycetes from soil samples at various sites along the Alar-Hotan desert highway in the Taklamakan desert. A total of 590 actinobacterial strains were recovered by the culture-dependent approach. Phylogenetic analysis based on 16S ribosomal RNA (rRNA) gene sequences unveiled a significant level of actinobacterial diversity with 55 genera distributed in 27 families of 12 orders. Thirty-six strains showed relatively low 16S rRNA similarities (<98.65%) with validly described species, among which four strains had already been characterized as novel taxa by our previous research. One hundred and forty-six actinobacterial isolates were selected as representatives to evaluate the antibacterial activities and mechanism of action by the paper-disk diffusion method and a double fluorescent protein reporter “pDualrep2” system, respectively. A total of 61 isolates exhibited antagonistic activity against the tested “ESKAPE” pathogens, among which seven strains could produce bioactive metabolites either to be able to block translation machinery or to induce SOS-response in the pDualrep2 system. Notably, *Saccharothrix* sp. 16Sb2-4, harboring a promising antibacterial potential with the mechanism of interfering with protein translation, was analyzed in detail to gain deeper insights into its bioactive metabolites. Through ultra-performance liquid chromatography (UPLC)-quadrupole time-of-flight (QToF)-MS/MS based molecular networking analysis and databases identification, four families of compounds (**1**–**16**) were putatively identified. Subsequent bioassay-guided separation resulted in purification of four 16-membered macrolide antibiotics, aldgamycin H (**8**), aldgamycin K (**9**), aldgamycin G (**10**), and swalpamycin B (**11**), and their structures were elucidated by HR-electrospray ionization source (ESI)-MS and NMR spectroscopy. All compounds **8**–**11** displayed antibacterial activities by inhibiting protein synthesis in the pDualrep2 system. In conclusion, this work demonstrates that Taklamakan desert is a potentially unique reservoir of versatile actinobacteria, which can be a promising source for discovery of novel species and diverse bioactive compounds.

## Introduction

The finding of novel bioactive compounds is a never-ending process, to meet the everlasting demand for novel drug with antimicrobial properties in order to combat escalating levels of antibiotic resistance in pathogenic microorganisms (Chokshi et al., 2019; Ardal et al., 2020). However, most of the large pharmaceutical and biopharmaceutical companies have terminated their anti-infective research programs due to high risk of failure and relatively low probability to achieve a tangible market success and profit. Until April 2020, there are 41 new antibiotics under global clinical development, but only 13 of them have the potential to treat pathogens on the WHO’s critical threat list (Pew Charitable Trusts, 2020). This fact has resulted in today’s eminent lack of new antibiotic drug leads, making us face a looming crisis that the emergence of resistance is outpacing the development of new antibiotics (Dickey et al., 2017; Woodworth et al., 2018). The previous admonitory terms of “gloom scenario,” “dark horizons,” and “back to the pre-antibiotic era” are becoming reality nowadays (Bravo et al., 2018). All these facts impel scientists to rapidly explore new chemical entities for developing novel antibiotics to target the emerging multidrug-resistant microbial pathogens that cause life-threatening infections.

Microorganisms, especially actinobacteria isolated from a diverse range of ecological niches, have been reported as the prolific producers of microbial bioactive secondary metabolites for pharmaceutical and agricultural applications, *etc*. The importance of these organisms is clearly seen from the fact that over 5,000 compounds have been reported from actinobacteria that contributed to the development of 90% of the commercial antibiotics being used for either clinical or research needs (Jose and Jha, 2016), such as Erythromycin, Gentamycin, Neomycin, Streptomycin, Chloramphenicol, Novobiocin, Teicoplanin, Vancomycin, Rifamycin, Chlortetracycline, *etc.* (Shah et al., 2017). However, after decades of exhausted excavation, the discovery of novel compounds from widely explored microbial strains is reaching a stagnation point, since it always yielded disappointing returns due to the frequent rediscovery of already known compounds. Thus, innovative drug discovery approaches are required in order to expedite the antibiotic discovery process.

One promising strategy is to selectively isolate, dereplicate, and screen representatives of novel and rare actinomycetes from neglected and unexplored habitats. The possibility of discovering novel bioactive molecules from extreme biomes could be greatly increased since harsh environmental conditions will give rise to novel actinobacteria with the capacity to synthesize novel metabolites (Goodfellow and Fiedler, 2010; Bull and Asenjo, 2013). As a result, bioprospecting actinobacteria from previously untapped sources, such as hyper-arid deserts, deep-sea sediments, permafrost soils, hydrothermal springs, *etc*. has been proposed as an important strategy to replenish the drug pipeline (Jose and Jha, 2016; Kolter and van Wezel, 2016). Deserts cover approximately 20% of the landmass on the planet (An et al., 2013), and the survival conditions in deserts are a huge challenge for microorganisms, as there is little available water and organic carbon, large temperature fluctuations, high exposure to UV irradiation, intense concentrations of metals and inorganic oxidants, and high salinity, pH in some areas (Koberl et al., 2011; Mohammadipanah and Wink, 2015). However, many surveys in recent years have revealed an extraordinary bacterial diversity across a range of desert environments, such as the Namibian desert (Wink et al., 2003), the Sahara desert (Zitouni et al., 2005; Meklat et al., 2011), the Mongolian desert (Kurapova et al., 2012), the Thar desert (Harwani, 2013; Tiwari et al., 2015), *etc*., and the most extensive studies are focused on the different sites in the Atacama desert in Northern Chile (Bull et al., 2016, 2018; Bull and Goodfellow, 2019). Furthermore, in the last decade, more than 30 novel natural products with diverse chemical structures and various biological activities were discovered from desert-derived actinobacteria, as exemplified by Chaxamycins A–D (Rateb et al., 2011a), Abenquines A–D (Schulz et al., 2011), Atacamycins A–C (Nachtigall et al., 2011), Chaxalactins A–C (Rateb et al., 2011b), Chaxapeptin (Elsayed et al., 2015), Lentzeosides A–F (Wichner et al., 2017), and Asenjonamides A–C (Abdelkader et al., 2018). These findings showed that the desert ecosystem inhabits a promising source of valuable actinobacterial species that can produce novel compounds with interesting chemical and pharmaceutical properties.

Covering as large as 346,905 km^2^ in the hinterland of the Tarim Basin in northwest China, the Taklamakan desert is known as the largest dunefield in China and the second largest drift desert in the world (Yang, 2018). It is categorized as harsh, hyper-arid, and continental climate (Yang et al., 2006) characterized by the extremely low water availability [very limited annual rainfall (10–50 mm/year)], strong evaporative potential (2,100–3,400 mm/year), large temperature fluctuations (the highest temperature in summer is 67.2°C and the lowest temperature in winter is −20°C), high ultraviolet radiation (3628.5 MJ/m^2^), poor organic carbon, and high osmotic stress in the soil or on the rock surfaces (Li et al., 2015; Jiang et al., 2019). These harsh living conditions in Taklamakan desert are a challenge for survival of microorganisms. However, a few reports, such as a finding (An et al., 2013) based on a metagenomic analysis of surface sand samples from Taklamakan desert indicated an unexpectedly large bacterial diversity residing in the harsh environment. To the best of our knowledge, up to the end of June 2020, a total of 19 novel species including one novel genus of actinobacteria have been reported from the Taklamakan desert. Our previous efforts to isolate endophytic actinobacteria from psammophytes in Taklamakan desert (Wang et al., 2020a) have successfully described a series of new species from genera, including *Prauserella*, *Nesterenkonia*, *Labedella*, and *Aeromicrobium* (Liu et al., 2015a,b; Li et al., 2019b,c). Although studies on the Chinese deserts are increasing in recent years, the exploration of the diversity of actinobacteria from the Taklamakan desert, to our knowledge, is still very limited. Furthermore, reports on the capability of actinobacteria to produce bioactive substances from Taklamakan desert are even rare. These facts demonstrate that the microbial ecology and their microbiota endowed with the potential to produce novel bioactive metabolites in Taklamakan desert need to be further studied.

In the present study, we continued an investigation on the biodiversity of cultivable actinobacteria sampled from eight arid soil samples along the Alar-Hotan desert highway in the Taklamakan desert and their antibacterial activity and capability to produce bioactive secondary metabolites were deeply explored. Meanwhile, a highlysensitive screening model, defined as double fluorescent protein reporter “pDualrep2” system, was implemented as a high-throughput screening model to distinguish the mechanism of action of bioactive metabolites secreted by actinobacterial strains. Finally, secondary metabolites of the *Saccharothrix* sp. 16Sb2-4 were examined in detail by analysis with comprehensive approaches, including hyphenated technique, MS/MS-based molecular networking analysis, databases identification, bioassay-guided separation, and NMR spectra analysis. Through this study, we wish to expand knowledge on the potential of actinobacteria residing in the Taklamakan desert, and attract more concerted efforts for discovery of new actinobacterial species and prominent antibiotic candidates from the harsh environment.

## Materials and Methods

### Site Description and Sample Collection

**Figure 1:**
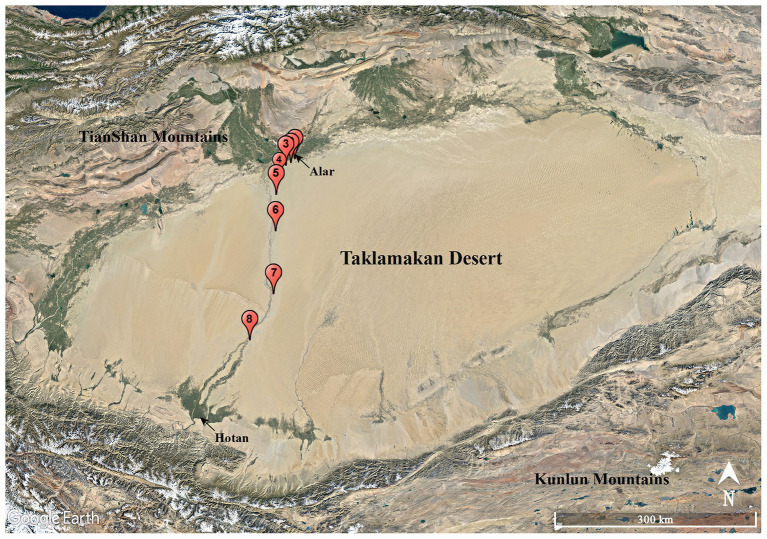
Geographic distribution of the sampling sites (S1–S8) along the Alar-Hotan desert highway in the Taklamakan desert, Xinjiang Uygur Autonomous Region, China.

A total of eight soil samples, coded as S1, S2, S3, S4, S5, S6, S7, and S8, were collected from various sites along the Alar-Hotan desert highway located in the Taklamakan desert, Xinjiang Uygur Autonomous Region, China in October 2016. The samples were collected at a depth of 10–15 cm from the surface. Sampling sites were at least 8 km away from each other as showed in Figure 1, and the sampling information in detail is listed in Supplementary Table S1. All the samples were collected into 50 ml labeled sterile Falcon tubes and stored at 4°C before transporting to our laboratory for further processing.

### Samples Processing and Isolation of Actinobacteria

Soil samples were first processed by air-dry at room temperature in the laminar flow hood for 8 h. Actinobacterial strains were isolated according to the standard serial dilution plating technique as described by Li et al. (2019a). Ten different isolation media supplemented with 1% (*v*/*v*) soil leaching liquor were prepared to isolate the actinobacterial strains (Supplementary Table S2). To inhibit the growth of Gram-negative bacteria and fungi, all media were supplemented with nalidixic acid, cycloheximide, and potassium dichromate to the final concentration of 20, 50, and 50 mg/l, respectively. The plates were incubated for 2–12 weeks at 30°C, and colonies that displayed differentiable morphologies were picked up from the original isolation plates and then sub-cultured on the Yeast-Malt Extract Agar (International Streptomyces Projects 2, ISP 2; Shirling and Gottlieb, 1966) plates to recover pure cultures with uniform colony morphology. The purified cultures were maintained on ISP 2 medium slants at 4°C and stored in 20% (*v/v*) glycerol suspensions at −80°C.

### Molecular Identification and Phylogenetic Analysis

Genomic DNAs for the 16S ribosomal RNA (rRNA) gene sequence analysis were extracted as described by Li et al. (2007). The 16S rRNA gene was amplified by PCR with two universal primers 27F (5'-AGAGTTTGATCMTGGCTCAG-3') and 1492R (5'-GGTTACCTTGTTACGACTT-3'), according to the method described by Liu et al. (2014). The PCR products were purified and then sequenced by an ABI PRISMTM 3730XL DNA Analyzer (Thermo Fisher Scientific, United States). The affiliation of the strains at genus-level was validated using the EzBioCloud’s Identify service (https://www.ezbiocloud.net/identify; Yoon et al., 2017) and the BLAST tool in GenBank NCBI database.^1^ The corresponding sequences of closely related type species were retrieved from the GenBank database. Multiple alignments were made using the Clustal_X tool in MEGA version 7.0 (Kumar et al., 2016). Phylogenetic tree based on the neighbor-joining algorithm (Saitou and Nei, 1987) was constructed under the Kimura’s two-parameter model (Kimura, 1980) with 1,000 bootstrap replicates. The 16S rRNA gene sequences obtained in this study were deposited in GenBank under the accession numbers: MT682401-MT682454, MT682461-MT682490, MT705160-MT705204, MH287062, MK787305, MH244160, and MK947033.

### Antibacterial Assay

Based on the phenotypic and phylogenetic analyses, 146 strains were selected to examine their antibacterial potentials. Each strain was transferred to three 500 ml Erlenmeyer flasks containing 100 ml of ISP 2 medium and then cultivated for 4–10 days at 30°C with rotated shaking at 180 rpm. The 300 ml fermentation broth for each isolate was centrifuged at 4,500 rpm for 20 min to separate the mycelium portion. The supernatants of fermentation broth were extracted twice with ethyl acetate (1:1, *v*/*v*) to get the organic layer and water layer. The whole organic layer and 50 ml water layer were dried up by rotary evaporation and dissolved in 3 ml methanol, respectively. The mycelium portion was soaked in acetone overnight, and then the leach liquor was concentrated under vacuum, and finally dissolved in 3 ml 50% methanol-water. Consequently, three kinds of samples were obtained from each strain for antibacterial assay by the paper-disk diffusion method. The methanol sample (60 μl) was dripped on a 6 mm paper disk in diameter. Besides, 60 μl methanol was used as the negative control, and levofloxacin solution (10 μl, 0.1 mg/ml) was used as the positive control. After dried up in the biosafety hood, the paper disks were transferred to agar plates with indicator bacteria and were incubated at 37°C for 24 h. Finally, the antibacterial activity was evaluated by measuring the diameters of the inhibition zone with a vernier caliper. The indicator bacteria used in antimicrobial assay were six sets of “ESKAPE” pathogenic bacteria, including *Enterococcus faecalis* (ATCC 33186 and 310682), *Staphylococcus aureus* (ATCC 29213 and ATCC 33591), *Klebsiella pneumonia* (ATCC 10031 and ATCC 700603), *Acinetobacter baumannii* (2799 and ATCC 19606), *Pseudomonas aeruginosa* (ATCC 27853 and 2774), and *Escherichia coli* (ATCC 25922 and ATCC 35218). Each set consisted of two strains, the former was drug-sensitive strain and the latter was drug-resistant strain. Strain 310682 was a clinical isolate resistant to vancomycin; Strain 2774 was a clinical isolate resistant to aminoglycosides and carbapenems. Indicator bacteria were obtained from American Type Culture Collection (ATCC) or the clinic and deposited in the Institute of Medicinal Biotechnology, Chinese Academy of Medical Sciences & Peking Union Medical College.

### Assay Based on Antibacterial Mechanism

A specific double fluorescent protein reporter system “pDualrep2” described previously was used to probe the mechanism action of compounds secreted by the antibacterial strains (Osterman et al., 2016). In brief, 100 μl ethyl acetate extract of each strain was dried up and then dissolved in 100 μl DMSO as the testing sample. About 2 μl of each sample solution was spotted on agar plates containing a lawn of the reporter strain *E. coli* JW5503. After overnight incubation at 37°C, the plate was scanned by ChemiDoc Imaging System (Bio-Rad Laboratories, United States) with two channels, “Cy3-blot” (553/574 nm, green pseudocolor) for red fluorescent protein (RFP) fluorescence, and “Cy5-blot” (588/633 nm, red pseudocolor) for Katushka2S fluorescence. Induction of expression of Katushka2S is triggered by translation inhibitors, while RFP is upregulated by DNA damage-induced SOS response. Levofloxacin (Lev, 50 μg/ml, 1 μl) and erythromycin (Ery, 5 mg/ml, 1 μl) were used as positive controls for inhibitors of DNA and protein biosynthesis, respectively.

### Large-Scale Fermentation and Extracts Preparation of *Saccharothrix* sp. 16Sb2-4

Based on results from antibacterial activity assay and preliminary mechanism detection, strain *Saccharothrix* sp. 16Sb2-4 with striking antibacterial activity was selected for large-scale fermentation and further chemical analysis. The strain was seeded in a 500 ml Erlenmeyer flask containing 100 ml of ISP 2 broth at 28°C for 2 days on a rotary shaker at 180 rpm. Then, 100 ml of seed culture was transferred into a 5 L Erlenmeyer flask containing 1 L of ISP 2 broth and fermented on a rotary shaker at 180 rpm at 28°C for 6 days. A total of 15 L fermented broth was collected and then centrifuged at 4,000 rpm at 25°C for 20 min. The cell-free supernatant was extracted three times with an equal volume of ethyl acetate. The organic phase was separated by a separatory funnel and then evaporated *in vacuo* to afford semi-solid crude extract (0.9 g). The extracted residue was suspended in methanol for next ultra-performance liquid chromatography (UPLC)-quadrupole time-of-flight (QToF)-MS/MS analysis and further chemical purification. Concomitantly, 1 L sterilized ISP 2 broth without addition of actinobacterial cells was extracted with ethyl acetate and evaporated to dryness parallelly, used as a medium control in metabolomic profiling of strain 16Sb2-4.

### UPLC-QToF-MS/MS Analysis

About 1 mg crude extract of strain 16Sb2-4 was dissolved in 1 ml methanol, and then was analyzed by UPLC coupled with QToF tandem mass spectrometry system (ACQUITY UPLC/Xevo G2-XS QTOF, Waters, United States) equipped with an electrospray ionization source (ESI). The sample (2 μl) was separated on the Waters ACQUITY UPLC BEH C18 column (2.1 × 100 mm, 1.7 μm) equipped with a Waters ACQUITY UPLC PDA detector at a flow rate of 0.3 ml/min. The column was eluted with a gradient mobile phase of acetonitrile-water containing 0.1% formic acid solution: 10% acetonitrile for 2 min, 10–90% acetonitrile for following 28 min, and finally, 90% acetonitrile for 3 min. The UV absorbance of the eluate was monitored by PDA detector from 190 to 400 nm. MS spectra were acquired by data-independent acquisition mode (MS^E^) for UNIFI analysis (Waters, United States) and data-dependent acquisition (DDA) for Global Natural Products Social Molecular Networking (GNPS) analysis, respectively. MS^E^ was carried out by operating the instrument at positive ion mode, applying the MS and MS/MS functions with 6 V low energy and 20–45 V high energy collision to collect the mass to charge ratio (*m/z*) from 100 to 2,500 Da. DDA was performed in positive ion mode, and the full MS survey scan was performed for 0.05 s time in the range of 100–2,500 Da, while MS/MS scanned over a mass range of 50–2,500 Da by the same scan time. The five most intense ions were further scanned for MS/MS fragmentation spectra. Data were collected and analyzed using the MassLynx V4.1 software (Waters, United States).

### Molecular Networking Analysis and Dereplication

The raw data obtained from DDA acquisition were used to create a molecular network. The DDA data were converted to the 32-bit mzML format using the MSConvert software (Chambers et al., 2012), and then uploaded on the GNPS web-platform (http://gnps.ucsd.edu; Wang et al., 2016a). The MS/MS molecular network was constructed using the “Classic” online workflow (METABOLOMICS-SNETS-V2) at GNPS. The precursor ion mass tolerance and MS/MS fragment ion tolerance were all set to 0.02 Da. A network was then created where edges were filtered to have a cosine score above 0.6 and more than three matched peaks. Further, edges between two nodes were kept in the network if and only if each of the nodes appeared in each other’s respective top 10 most similar nodes. The spectra in the network were then searched against GNPS spectral libraries to annotate and identify metabolites through the database search of MS/MS spectra. The library spectra were filtered in the same manner as the input data. The molecular network was visualized using Cytoscape software v3.8.0 (Shannon et al., 2003). To improve the dereplication, a manual annotation was conducted in UNIFI informatics platform (Waters, United States) by searching the microbial natural products database, The Natural Products Atlas (www.npatlas.org; Van Santen et al., 2019) based on the predicted accurate mass value. Sample of the medium control was analyzed in the same procedure as above.

### Isolation and Identification of Bioactive Metabolites Produced by *Saccharothrix* sp. 16Sb2-4

To trace the bioactive metabolites produced by *Saccharothrix* sp. 16Sb2-4, bioassay-guided fractionation was performed with the methicillin-resistant *S. aureus* (MRSA) ATCC 33591 as the indicator bacterium. The crude extract dissolved in methanol was subjected to Sephadex LH-20 column chromatography with elution of methanol to obtain different fractions. Each fraction was screening for antibacterial activity against the indicator bacterium. The bioactive fractions were merged and further subjected to flash chromatography (Biotage, United States) using a reversed-phase C18 column with a gradient elution of 20, 40, 60, 70, and 80% (*v/v*) MeOH/H_2_O mixtures to yield 10 subfractions (subfrs. 1–10). Each subfraction was assayed by anti-MRSA activity, and the bioactive subfractions (subfrs. 5–8) were analyzed by UPLC-MS/MS to seek compounds of interest. According to the UPLC-MS/MS results, the bioactive subfractions with targeted compounds were combined and further purified by semi-preparative high-performance liquid chromatography (HPLC, Agilent 1200, Agilent Technologies Inc., United States) equipped with an Agilent ZORBAX SB-C18 column (9.4 × 250 mm, 5 μm). Subfrs. 5–6 were combined and purified by HPLC (solvent, 50% MeOH in H_2_O; flow rate, 2.0 ml/min; detection, UV 254 nm) to yield compounds **8** (7.8 mg, *t*_R_ = 43 min) and **9** (3.1 mg, *t*_R_ = 47 min). Subfrs. 7–8 were merged and purified by HPLC (solvent, 58% MeOH in H_2_O; flow rate, 2.0 ml/min; detection, UV 254 nm) to afford compounds **10** (4.6 mg, *t*_R_ = 41 min) and **11** (5.3 mg, *t*_R_ = 43 min).

The structures of four metabolites were determined by the analysis of HR-ESI-MS, ^1^H and ^13^C NMR data. The HR-ESI-MS data were recorded on a Xevo G2-XS QTof mass spectrometer (Waters, United States). The ^1^H and ^13^C NMR spectral data were recorded on the Brucker Avance III 600 (600 MHz) spectrometer (Bruker, Germany). The purified compounds were dissolved in chloroform-*d*/methanol-*d_4_*, and the residual solvent signals were used for referencing spectra in ^1^H and ^13^C dimensions (Gottlieb et al., 1997).

## Results

### Biodiversity of Cultivable Actinobacteria Derived From Soil Samples of Taklamakan Desert

**Figure 2:**
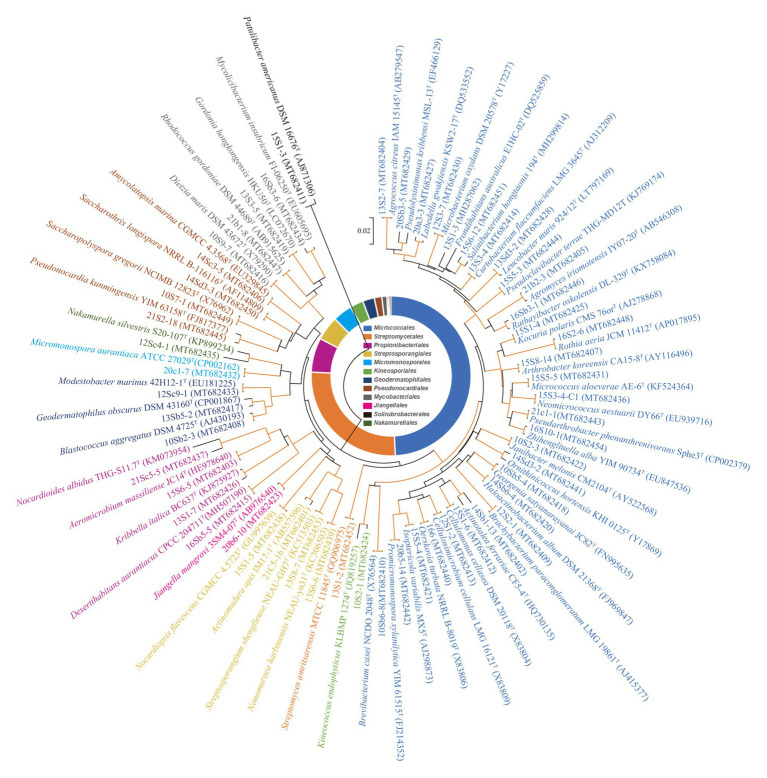
Phylogenetic tree based on 16S ribosomal RNA (rRNA) genes using neighbor-joining method with 1,000 bootstrap replications for 55 representative actinobacterial strains and their closely related type strains. Red lines indicate bootstrap support values >70% for the branch. Strains in the same color belong to the same order. Bar, 2 nt substitutions per 100 nt.

A total of 860 strains were isolated from the eight soil samples collected in the Taklamakan desert. The phylogeny of the strains was evaluated based on the comparative analysis of their partial 16S rRNA gene sequences (approximately 800 bp) in EzBioCloud database. The results showed 590 out of 860 isolates were confirmed as actinobacterial strains and assigned to 55 genera in 27 families of 12 orders (Supplementary Table S3), which was further supported by analysis of their 16S rRNA gene sequence-based dendrogram (Figure 2). Analyzing the relative abundance at the order level, nearly half of the 590 actinobacterial strains were affiliated to *Micrococcales* (49.2%, 290 strains), followed by *Streptomycetales* (26.7%, 157 strains), and *Propionibacteriales* (6.9%, 41 strains). The others belonged to order *Streptosporangiales* (29 strains), *Micromonosporales* (23 strains), *Kineosporiales* (16 strains), *Geodermatophilales* (14 strains), *Pseudonocardiales* (9 strains), *Mycobacteriales* (6 strains), *Jiangellales* (2 strains), *Solirubrobacterales* (2 strains), and *Nakamurellales* (1 strain). At the genus level, the predominant genus was *Streptomyces* (26.7%, 157 strains), and among the rest 433 non-*Streptomyces* strains, the dominant genus was *Microbacterium* (16.1%, 95 strains), followed by *Aeromicrobium* (6.4%, 38 strains), *Kocuria* (4.9%, 29 strains), *Nocardiopsis* (4.4%, 26 strains), and *Brachybacterium* (4.4%, 26 strains).

**Figure 3:**
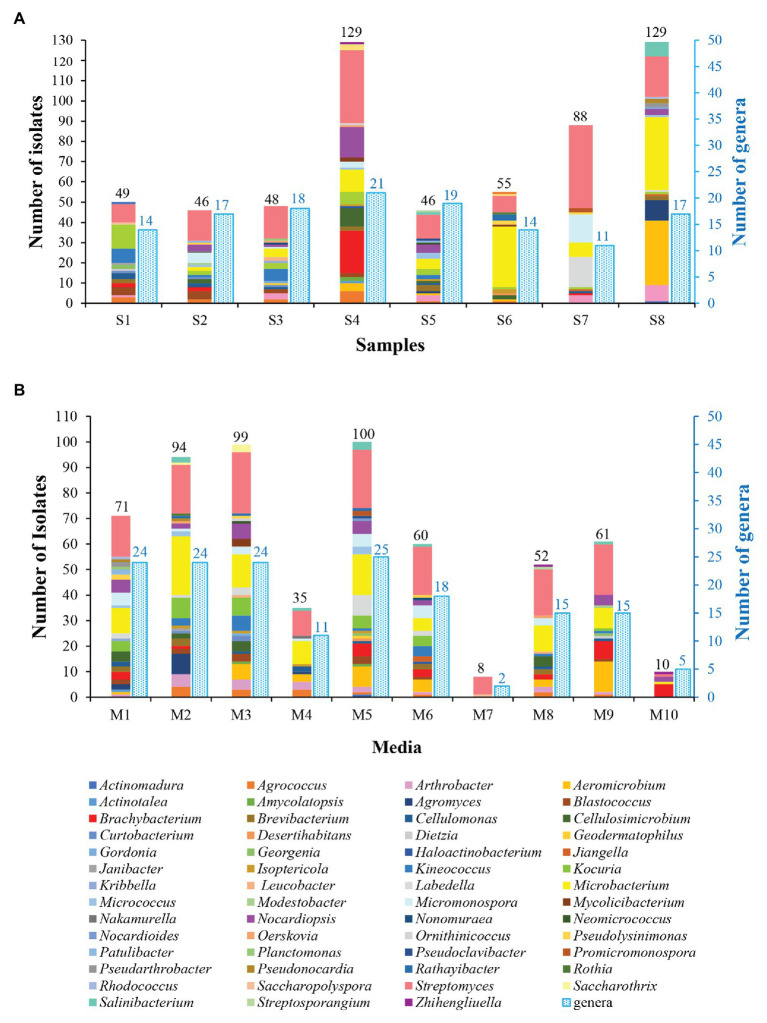
Diversity of cultivable actinobacteria isolated from soil samples collected in Taklamakan desert. **(A)** Actinobacterial isolates recovered from different sampling sites. **(B)** Actinobacterial isolates recovered from the different culture media.

The genera distribution of 590 actinobacterial strains in eight samples and 10 media are displayed in Figure 3. Sample 4 gave the highest diversity (21 genera), followed by sample 5 (19 genera), sample 3 (18 genera), samples 2 and 8 (17 genera), samples 1 and 6 (14 genera), and sample 7 (11 genera). The isolation media played a major influence on the diversity of isolates recovered. The CMKA medium (Lai et al., 2014; M5) was the most effective in terms of the number and diversity of isolates obtained (100 strains distributed in 25 genera); three different isolation media, including modified Gauze’s NO. 1 synthetic medium (M1), ISP 2 (M2), and R2A (M3) media also resulted in relatively efficient isolations, and 24 genera were retrieved by each of media above; meanwhile, 18, 15, 15, and 11 genera were obtained from Raffinose-Histidine medium (M6), Proline medium (M8), Casein-Glucose medium (M9), and Modified Cellulose-Casein medium (M4), respectively. Trehalose-Proline medium (M7) yielded the lowest diversity with only eight strains distributed in two genera (*Streptomyces* and *Saccharopolyspora*). The high-salt medium (M10) was used to isolate halophilic or halotolerant strains in desert samples, and 10 strains belonged to five genera (*Brachybacterium*, *Nocardiopsis*, *Streptomyces*, *Microbacterium*, and *Zhihengliuella*) were isolated from this medium. Among the 55 genera of actinobacteria, *Streptomyces* spp. seemed to be the most abundant since they could be recovered in all of the isolated media from all of the samples.

### Novelty of Cultivable Actinobacteria

Based on comparative results with EzBiocloud database, 36 strains exhibited less than 98.65% similarities (the threshold for differentiating two species Kim et al., 2014) in 16S rRNA gene sequences with validly described species (Supplementary Table S4), indicating that these isolates could be regarded as primary candidates for novel taxon. These 36 putative novel isolates were preliminarily affiliated into 12 families, including *Microbacteriaceae* (10 strains), *Dermabacteraceae* (5 strains), *Promicromonosporaceae* (3 strains), *Cellulomonadaceae* (3 strains), *Nocardioidaceae* (3 strains), *Nocardiopsaceae* (3 strains), *Streptomycetaceae* (3 strains), *Kineosporiaceae* (2 strains), *Bogoriellaceae* (1 strain), *Geodermatophilaceae* (1 strain), *Nakamurellaceae* (1 strain), and *Patulibacteraceae* (1 strain). Notably, 10 putative novel isolates showed close relationships with members of family *Microbacteriaceae*. Pairwise comparison of 16S rRNA gene sequences from the 10 isolates showed they shared the highest sequence identities of 97.29–98.44% to the closest recognized species of family *Microbacteriaceae*. Further phylogenetic analysis of the 10 *Microbacteriaceae*-like isolates based on the neighbor-joining tree is shown in Supplementary Figure S1. Phylogenetic analysis indicated that these isolates were diversely distributed within the family *Microbacteriaceae* and formed distinct clusters within the neighbor-joining tree. Isolate 15S1-1, sharing the highest 16S rRNA gene similarity of 97.95% to the type strain of *Agromyces arachidis*, formed a distinct branch within the lineage of the genus *Agromyces*, suggesting that isolate 15S1-1 might represent a novel species of the genus *Agromyces*. Isolates 20Sb5-7 and 16Sc5-2 formed a statistically well supported cluster with one another and fell into a coherent subclade with *Microbacterium wangchenii* dk512^T^ within the *Microbacterium* group. The 16S rRNA gene sequences of 20Sb5-7 and 16Sc5-2, respectively showed 98.16 and 98.44% identities to the nearest neighbor *M. wangchenii* dk512^T^, indicating that the two isolates might be identified as new species of the genus *Microbacterium*. Four isolates, 21Sb5-5, 21Sc5-12, 21Sb2-13, and 15S6-12, showing the highest similarity values of 97.76–98.15% to *Salinibacterium hongtaonis* 194^T^, gathered in one monophyletic cluster that was independent from other subclusters corresponding to the established genera of the family *Microbacteriaceae*, indicating that they might represent novel species of a new genus. Similarly, strains 20Sb3-5 and 16Sc1-5 formed a distinct monophyletic clade within the family *Microbacteriaceae* and they showed the highest 16S rRNA gene similarities, 98.13 and 98.16%, to the closest type strain of *Pseudolysinimonas kribbensis*, suggesting that isolates 20Sb3-5 and 16Sc1-5 might belong to a new genus. Above nine putative novel species will be further characterized by the polyphasic approach to determine their taxonomic positions. In addition, strain 13S1-3 has been characterized as a novel species of a new genus in the family *Microbacteriacea* by our polyphasic taxonomic analyses with the proposed name “*Planctomonas deserti*” (Liu et al., 2019a). Besides the 10 isolates in family *Microbacteriaceae*, three isolates were characterized as type species of new taxa based on our previous study of polyphasic taxonomy. Strain 12Sc4-1 has been characterized as a new species of the genus *Nakamurella* with the proposed name *Nakamurella deserti* (Liu et al., 2019b); strain 21Sc5-5 has been characterized as a new species of the genus *Nocardioides* with the proposed name *Nocardioides vastitatis* (Liu et al., 2020a); and strain 16Sb5-5 has been characterized as a new species of the genus *Desertihabitans* with the proposed name *Desertihabitans brevis* (Liu et al., 2020b). Notably, although the similarity of 16S rRNA gene sequence between 16Sb5-5 and its closest neighbor *Desertihabitans aurantiacus* CPCC 204711^T^ was as high as 99.6%, the average nucleotide identity (ANI) and *in silico* DNA-DNA hybridization (DDH) values were all less than the widely accepted thresholds to distinguish two species [95% for ANI (Richter and Rossello-Mora, 2009) and 70% for DDH (Chun et al., 2018)]. Therefore, strain 16Sb5-5^T^ has been proved as a novel species of the genus *Desertihabitans* (May and Grabowicz, 2018). Phylogenetic analysis of the other 24 potential novel strains based on the neighbor-joining trees constructed with 16S rRNA gene sequences are shown in Supplementary Figures S2A–J. The presence of these rare actinobacteria emphasizes the unique microbial diversity characteristics of the Taklamakan desert and supports the idea of extreme ecological environments being an important resource for novel species and chemical entities.

### Antibacterial Activity

**Figure 4:**
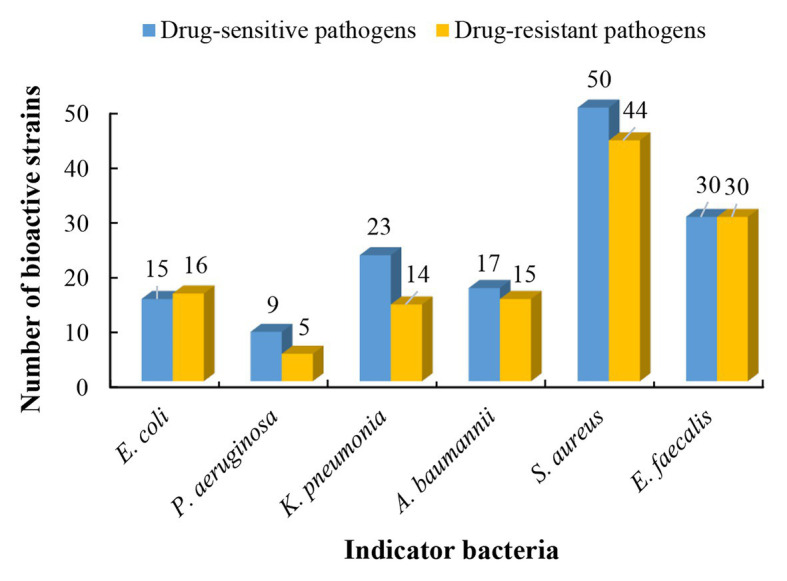
The antibacterial profile of the desert-derived actinobacteria against “ESKAPE” pathogens.

Based on results of phylogenetic and morphological analyses, 146 actinobacterial isolates affiliated to 55 different genera were selected as representatives to evaluate the antimicrobial potential against a panel of “ESKAPE” bacteria. Among the 146 tested isolates, 61 strains (41.8%) exhibited antagonistic activity against at least one of the tested pathogens (Supplementary Table S5). The 61 antibacterial strains were affiliated to 19 genera, including *Streptomyces* (37 strains), *Nocardiopsis* (3 strains), *Micromonospora* (3 strains), *Cellulosimicrobium* (2 strains), *Microbacterium* (2 strains), *Saccharothrix* (2 strains), *Actinomadura* (1 strain), *Aeromicrobium* (1 strain), *Blastococcus* (1 strain), *Cellulomonas* (1 strain), *Planctomonas* (1 strain), *Janibacter* (1 strain), *Kineococcus* (1 strain), *Kocuria* (1 strain), *Leucobacter* (1 strain), *Pseudolysinimonas* (1 strain), *Desertihabitans* (1 strain), and *Pseudonocardia* (1 strain). The antimicrobial profile of the actinobacteria against different pathogenic bacteria was shown in Figure 4. Out of the 61 antimicrobial isolates, 25 isolates exhibited antagonistic activity against both Gram-negative and Gram-positive bacteria; 29 isolates exhibited antibacterial activity against only Gram-positive bacteria; and seven isolates against only Gram-negative bacteria. Regarding the drug-sensitive pathogens tested, inhibitory zones were observed most frequently against the Gram-positive bacteria such as *S. aureus* (50 isolates) and *E. faecalis* (30 isolates), followed by Gram-negative bacteria, such as *K. pneumonia* (23 isolates), *A. baumannii* (17 isolates), *E. coli* (15 isolates), and *P. aeruginosa* (9 isolates). Concerning the drug-resistant pathogens tested, activity against Gram-positive bacteria remained the most frequent where 44 of the 146 tested strains presented inhibitory zones against MRSA and 30 strains against vancomycin-resistant *E. faecalis* (VRE). The activity against the Gram-negative bacterium *P. aeruginosa* was still the least frequent (five isolates), and 16, 15, and 14 isolates were active against *E. coli*, *A. baumannii*, and *K. pneumonia*, respectively. This phenomenon could be attributed to the sophisticated outer membrane and multiple efflux pumps possessed by Gram-negative bacteria, which served as a robust permeability barrier for preventing many antibiotics from reaching their intracellular targets (Miller, 2016; May and Grabowicz, 2018). Five isolates, including *Nocardiopsis* sp. 14Sc5-11, *Saccharothrix* sp. 16Sb2-4, *Microbacterium* sp. 14Sc5-17, and *Streptomyces* sp. 14Sb4-3 and 13S9-1, appeared to have a broad spectrum of antimicrobial activity against at least 11 test strains. Among them, *Saccharothrix* sp. 16Sb2-4 showed the highest inhibitory activities against VRE and methicillin-sensitive *S. aureus* (MSSA) with inhibition zones 24 and 23 mm in diameter, respectively. Notably, in some cases, only one type of extract demonstrated activity against one or some indicator pathogens, for example, antibacterial activity was observed only from ethyl acetate extracts of cultural broth of *Streptomyces* sp. 15S4-1, 10Sb5-5, and 10S9-4. On the contrary, only mycelia extracts of *Nocardiopsis* sp. 12Sb1-6, *Micromonospora* sp. 12Sd5-1, and *Streptomyces* sp. 12S1-1 showed antibacterial activities against *P. aeruginosa*.

### Antibacterial Mechanism Assay

**Figure 5:**
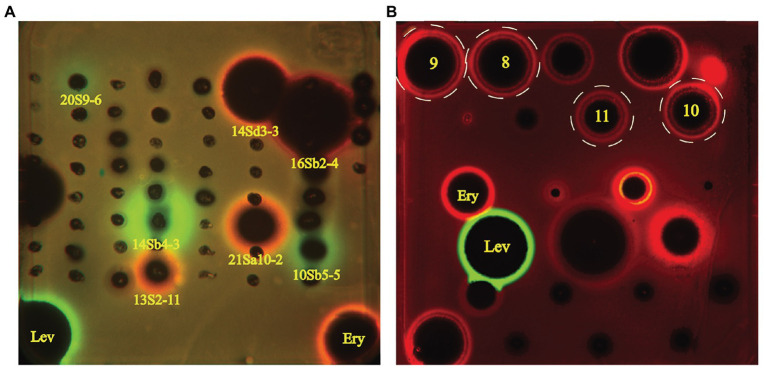
Induction of two-color dual reporters in pDualrep2 system by inhibitors of the ribosome progression or inhibitors of DNA replication, respectively. Spots of erythromycin (Ery), levofloxacin (Lev), and tested samples were placed on the surface of an agar plate containing *Escherichia coli ΔtolC* cells transformed with the pDualrep2 reporter plasmid. Shown is the fluorescence of the lawn of *E. coli* cells scanned at 553/574 nm (green pseudocolor) for red fluorescent protein (RFP) fluorescence and 588/633 nm (red pseudocolor) for Katushka2S fluorescence. Induction of expression of Katushka2S is triggered by translation inhibitors, while RFP is upregulated by induction of DNA damage SOS response. **(A)** Screening results of crude extracts of 61 strains; **(B)** Evaluation of compounds **8–11** (indicated in white dotted circles) isolated from *Saccharothrix* strain 16Sb2-4.

Sixty-one desert-derived actinobacterial strains are potential candidates to produce antibiotics with different mechanisms of action. To distinguish strains with different antibacterial mechanisms, ethyl acetate extracts from culture broth of 61 bioactive strains were screened by the double fluorescent protein reporter “pDualrep2” system, which is a highlysensitive screening model for probe of compounds that inhibit protein translation or DNA biosynthesis (Osterman et al., 2016). The screening results are shown in Figure 5A. Four strains, including two *Streptomyces* sp. (13S2-11 and 21Sa10-2) and two *Saccharothrix* sp. (14Sd3-3 and 16Sb2-4) could induce expression of far-RFP reporter Katushka2S, acting as typical inhibitors of protein translation as the erythromycin did. Meanwhile, three *Streptomyces* strains (14Sb4-3, 10Sb5-5, and 20S9-6) could induce expression of RFP reporter, triggering DNA damage-induced SOS response as the levofloxacin did.

### Metabolites Identification of *Saccharothrix* sp. 16Sb2-4 Using MS/MS-Based Molecular Networking

In order to highlight antimicrobial potential of the desert-derived isolates, analysis of the metabolites should perform with a potent antimicrobial metabolite-producing isolate. Strain 16Sb2-4 in genus *Saccharothrix* showed a promising inhibitory activity against both Gram-positive and Gram-negative “ESKAPE” pathogens, especially multi-drug resistant (MDR) bacteria, such as *P. aeruginosa* (16.8 mm), *A. baumannii* (13 mm), and *K. pneumoniae* (10.7 mm), MRSA (22.8 mm), and VRE (24.2 mm). Meanwhile, the strain exhibited activity to block translation machinery in the pDualrep2 system. Blast analysis based on the 16S rRNA gene sequence showed strain 16Sb2-4 shared the highest nucleotide sequence similarity (99.7%) with *Saccharothrix xinjiangensis* NBRC 101911^T^, a strain that was previously isolated from Tianchi Lake, Xinjiang Uygur Autonomous Region, China (Hu et al., 2004). Strain of *S. xinjiangensis* has ever been reported to produce a range of bioactive compounds, such as the tianchimycins A and B (Wang et al., 2013), cyanogriside I and J, caerulomycin A and F (Lahoum et al., 2019), and caerulomycin M and saccharopyrone (Babadi et al., 2020). Taking these facts into consideration, strain 16Sb2-4 was prioritized to conduct a chemical analysis to gain deeper insights into its bioactive metabolites.

Ultra-performance liquid chromatography coupled with QToF-HR-MS can offer a high degree of mass resolution, sensitivity, and accuracy for identification of chemical components, especially for trace level ingredients in complex natural product extracts (Kaufmann, 2014; Deng et al., 2016). Therefore, an UPLC-QToF-MS/MS based untargeted metabolite profiling was carried out for a comprehensive understanding of chemical diversity in the cultural broth of strain 16Sb2-4. GNPS platform was employed to detect the MS/MS structural relatedness among molecules in an automated manner; then the software generated a molecular network wherein molecules with related scaffolds clustered together (Wang et al., 2016a). A MN-based network representing the ions detected in the crude extract of *Saccharothrix* sp. 16Sb2–4 was constructed, revealing 981 nodes representing unique spectra in total, of which 600 nodes were clustered in 80 spectral families conformed with at least 2 nodes (Supplementary Figure S3). Not all the network nodes correspond to a single molecule since some nodes represent adducts.

**Figure 6:**
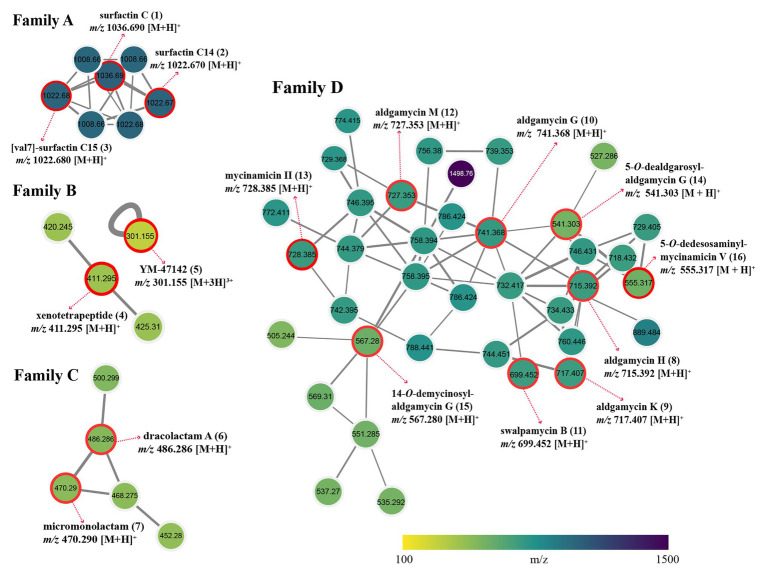
Four identified metabolite families in molecular network of strain *Saccharothrix* sp. 16Sb2-4. Each node represents *m/z* value of the parent ion and the edge thickness signifies cosine score similarity. Family A, surfactin lipopeptides; Family B, cyclic depsipeptides; Family C, polyene macrolactams; and Family D, 16-membered macrolides.

Global Natural Products Social Molecular Networking dereplication based on matching with its MS/MS spectral database allowed annotation of two families of potent bioactive compounds (Family A and B, Figure 6). Family A was identified as known lipopeptides, including surfactin C (**1**), surfactin C14 (**2**), and [val7]-surfactin C15 (**3**); Family B was annotated as cyclic depsipeptides including xenotetrapeptide (**4**) and YM-47142 (**5**). Detailed information for these annotated peak ions to known metabolites is displayed in Supplementary Table S6. The surfactins are cyclic lipopeptides produced by multiple genera of bacteria, such as *Bacillus*, *Streptomyces*, *Micromonospora*, and *Pseudomonas*. This chemical class is not only regarded as a powerful lipopeptide biosurfactant, but also acts as effective antibiotic agents due to the ability to penetrate cell membranes of Gram-positive and Gram-negative bacteria, as well as fungi (Seydlová and Svobodová, 2008; Meena and Kanwar, 2015). The cyclic depsipeptide YM-47142 was isolated from the fermented broth of a *Flexibacter* bacterium as a potential inhibitor of human leucocyte elastase (HLE) enzyme, which involved in the pathogenesis of a variety of inflammatory diseases, such as emphysema, acute respiratory distress syndrome, and rheumatoid arthritis (Orita et al., 1995). Xenotetrapeptide was a NRPS-derived cyclic tetrapeptide produced by *Xenorhabdus nematophila*, but its activities have not yet to be characterized (Kegler et al., 2014). In addition, some synthetic contaminants such as phthalates (dibutyl phthalate and dioctyl phthalate) commonly occur from plasticware used in experiments were also detected; some dubious compounds readily formed from medium components, such as dipeptides (Ile-Tyr and Tyr-Pro), diketopiperazines [cyclo (Phe-Pro), cyclo (Val-Phe), cyclo (Leu-Phe), and cyclo (Leu-Pro)], and fatty acids (Conjugated linoleic acid and 9-Octadecenamide) were also found in metabolomic profiling of media controls (Supplementary Figure S3). These non-metabolites were easy to be precluded through GNPS dereplication.

Many nodes in crude extract of *Saccharothrix* sp. 16Sb2-4 did not match with the large datasets of the GNPS repository, indicating the putative presence of new metabolites or the absence of similar compounds from the GNPS repository. Therefore, an additional manual dereplication was conducted in UNIFI informatics platform by search of the spectral data against the bacterial and fungal natural products database, the Natural Products Atlas (www.npatlas.org; Van Santen et al., 2019). The annotation of these compounds was supported by comparison of the precursor ion *m/z* values, fragmentation pattern, and UV spectra with reference data. The manual dereplication allowed annotation of another two families of compounds presented in the molecular network (Family C and D, Figure 6). The Family C subnetwork containing five spectral nodes was identified as the cluster of macrolactam compounds. Two known 22-membered polyene macrolactams, dracolactam A (**6**) and its biosynthetic precursor micromonolactam (**7**), were annotated in the extract of the strain. Meanwhile, another three structurally related nodes [*m/z* 452.280, 468.275, and 500.299 (M+H)^+^] identified as derivatives of dracolactam can be found in this molecular cluster. Although no bioactivities were reported for dracolactam (Hoshino et al., 2017) and micromonolactam (Skellam et al., 2013), in the previous studies, it is worthy to concern this class of derivatives due to some polyene macrolactams were reported to display antibacterial, antiprotozoal, or cytotoxic activities (Skellam et al., 2013; Hoshino et al., 2017; Lim et al., 2018). The Family D subnetwork containing 38 spectral nodes with a strong spectral similarity score (cosine score > 0.6) was annotated as the cluster of the 16-membered macrolides. Nine compounds belonging to the 16-membered macrolide family were tentatively identified, *viz*. aldgamycin H (**8**), aldgamycin K (**9**), aldgamycin G (**10**), swalpamycin B (**11**), aldgamycin M (**12**), mycinamicin II (**13**), as well as three deglycosylated derivatives, 5-*O*-dealdgarosyl-aldgamycin G (**14**), 14-*O*-demycinosyl-aldgamycin G (**15**), and 5-*O*-dedesosaminyl-mycinamicin V (**16**). Annotation of these diagnostic 16-membered macrolide antibiotics is of significance, as this class of antibiotics is well-known inhibitor of bacterial protein synthesis, and characterized as among the safest antibacterial reagents in wide clinical use. Many macrolides have successfully been used to treat infections caused by Gram-positive organisms, certain Gram-negative and anaerobic bacteria (Elshahawi et al., 2015; Tang et al., 2016; Arsic et al., 2018; Karpinski, 2019). This finding further emphasized to continue our effort on targeted isolation, identification and activity evaluation of these putative 16-membered macrolides.

### Bioactive Macrolides Produced by *Saccharothrix* sp. 16Sb2-4

**Figure 7:**
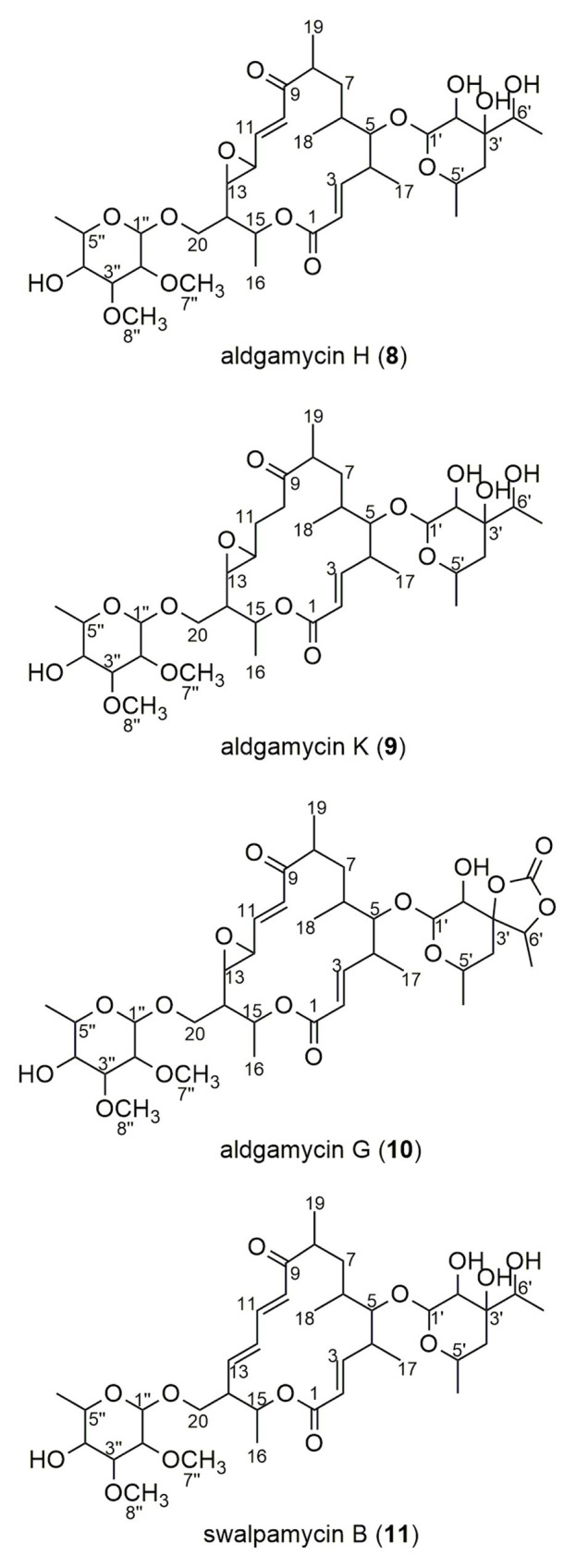
Chemical structures of compounds **8–11** isolated from cultural broth of *Saccharothrix* sp. 16Sb2-4.

The crude extract from large-scale fermentation of strain 16Sb2-4 was successively fractionated by Sephadex LH-20 column chromatography, reversed-phase C18 column chromatography, and semi-preparative HPLC. Bioassay against MRSA coupling with UPLC-MS/MS analysis was used to monitor the presences of bioactive 16-membered macrolide antibiotics. Four targeted compounds (**8–11**) were obtained and their structures were determined using HR-ESI-MS, NMR (Supplementary Figures S4–S11; Supplementary Table S7) as well as comparison with spectral data from the literatures. As suggested by molecular networking analysis, the four compounds were indeed afore-mentioned 16-membered macrolides, *viz*. aldgamycin H (**8**; Zitouni et al., 2007), aldgamycin K (**9**; Wang et al., 2016b), aldgamycin G (**10**; Mizobuchi et al., 1986; Zitouni et al., 2007), and swalpamycin B (**11**; Zitouni et al., 2007; Wang et al., 2013), as shown their structures in Figure 7. The antibacterial mechanism of compounds **8–11** was evaluated by the pDualrep2 system. As shown in Figure 5B, compounds **8–11** could induce Katushka2S expression, exerting their antibacterial effects by inhibiting protein synthesis. This finding was corresponding to the preliminary screening result of the crude extract of strain 16Sb2-4 (Figure 5A). Given the fact that compounds **8–11** were main bioactive metabolites isolated under guidance of anti-MRSA activities, the activity against Gram-positive bacteria in the cultural broth of strain 16Sb2-4 can be explained mostly by the accumulation of these macrolide antibiotics. In addition, some additional nodes that were structurally related identified aldgamycins in the molecular network but could not be assigned to any of the previously reported compounds, suggesting the potential of strain 16Sb2-4 for producing novel bioactive metabolites. Further purification and structural elucidation of these new putative compounds are still ongoing.

## Discussion

Deserts are thought to possess relatively harsh environments that are still poorly explored and waiting for more scientific interventions to search for pharmaceutical microorganisms. Many previous studies have suggested *Actinobacteria* are the major taxa in the phylum-level composition of bacteria in many arid desert soil (Mohammadipanah and Wink, 2015), as exemplified by the Atacama Desert (Crits-Christoph et al., 2013), where *Actinobacteria* were the most dominant phylum (72–88%). Analysis of bacterial diversity of sandy arid soil in southeast Morocco revealed that *Actinobacteria* were the most frequent groups (57%; Gommeaux et al., 2010). The bacterial communities in Namib Desert soils were dominated by the phylum *Actinobacteria* (49%; Makhalanyane et al., 2013). In some arid areas, *Actinobacteria* are among the three most abundant phyla (usually along with the *Firmicutes* and *Proteobacteria*), such as the desert soil of Aridic Calcisols in Kazakhstan (Lynch et al., 2014), a shrub root zone of desert (Steven et al., 2012), and a high elevation desert (Lynch et al., 2014). This is unsurprising, since actinobacteria were proved to possess unique capacity for sporulation, wide metabolic and degradative capacity, competitive advantages *via* secondary metabolite synthesis, and multiple UV repair mechanisms. (McCarthy and Williams, 1992; Chater and Chandra, 2006; Gao and Garcia-Pichel, 2011; Makhalanyane et al., 2015).

Although several pilot studies have been carried out to investigate the bacterial diversity in the Taklamakan desert, the knowledge of its microbial communities and the potential of microbiota to produce bioactive metabolites is still little and patchy. Bao et al. (2011) investigated bacterial diversity of soils collected from *Populus Euphratica* forest in the hinterland of Taklamakan desert. Twenty-seven strains obtained fell into 13 genera in four bacterial phyla, in which 16 strains were actinobacteria with *Streptomyces* and *Kocuria* as the dominant genera. All isolates demonstrated certain enzymatic activity which can be used as inoculants for silage and biofertilizer. Dong et al. (2013) explored the diversity and bioactivity of actinomycetes at the south edge of the Taklamakan desert. One hundred and sixty-five actinobacterial strains were isolated, and they distributed in 24 different genera in 16 families with the dominant genera *Streptomyces* and *Nocardiopsis*. Meanwhile, nearly two thirds strains exhibited antifungal or antibacterial activities. Yu et al. (2015) isolated 52 strains of radiation-resistant bacteria from the soil sample of Taklamakan desert, in which 32 strains were affiliated into phylum *Actinobacteria* including the genera *Agrococcus*, *Arthrobacter*, *Cellulomonas*, *Kocuria*, *Knoella*, and *Nocardioides*. All strains were found to possess reactive oxygen species (ROS)-scavenging enzymes to protect cells against oxidative damage and ionizing radiation. In our recent publication, 320 endophytic actinobacterial strains, assigned to 23 genera in 14 families, were isolated from psammophytes collected in Taklamakan desert with *Streptomyces* as the dominant genus (Wang et al., 2020a). In the present study, a considerable diversity of cultivable actinobacteria was obtained from eight different soil samples along the Alar-Hotan desert highway in the Taklamakan desert. A total of 590 actinobacterial strains were assigned to 55 genera in 27 families of 12 orders, suggesting that enormous actinobacterial strains are widespread throughout this arid region. Comparing with previous studies, this is the first time to recover such magnitude of diversity of cultivable actinobacteria from the Taklamakan desert merely in eight soil samples. Notably, genera *Pseudarthrobacter*, *Oerskovia*, *Patulibacter*, and *Neomicrococcus*, to our knowledge, are the first time to be detected from the desert ecosystem.

*Streptomyces* species were dominantly isolated from the Taklamakan desert soil, which is congruent with many previous studies in desert ecosystems worldwide (Hozzein et al., 2008; Okoro et al., 2009; Selvameenal et al., 2009; Tiwari et al., 2015). Apart from the ubiquitous genus *Streptomyces*, some rare genera found in this study can be classified as indigenous desert types, as they have been often detected in desert-like environments (arid environments). For example, members of the family *Geodermatophilaceae*, notably the genera *Geodermatophilus*, *Blastococcus*, and *Modestobacter* are known to be resistant to desiccation, low nutrition, strong ionizing radiation, UV-light, and heavy metals (Sghaier et al., 2016). Members of *Actinomadura*, *Micromonospora*, and *Streptosporangium* are thermotolerant species widespread in desert soils (Kurapova et al., 2012). Species of *Gordonia*, *Nocardioides*, and *Amycolatopsis* are reported to be desiccation-resistant (Mohammadipanah and Wink, 2015); *Micrococcus*, *Brachybacterium*, *Nocardia*, *Microbacterium*, *Kocuria*, *Kineococcus*, and *Micrococcus* are reported to be radiation-resistant (Mohammadipanah and Wink, 2015; Yu et al., 2015; Sayed et al., 2020). Genera *Nocardiopsis*, *Haloactinobacterium*, *Kocuria*, *Saccharopolyspora*, and *Nesterenkonia* are mostly halotolerant or halophilic actinomycetes isolated from salty desert environment (Hamedi et al., 2013). Deriving of these unique genera of actinobacteria not only demonstrates the rich actinobacterial diversity in Taklamakan desert but also provides more desert-derived strains for further study on their potential in medicine and biological function in ecology.

In the antibacterial assay, 61 isolates affiliated to 19 genera exhibited antagonistic activity against the tested “ESKAPE” pathogens. Analysis of composition of 61 positive strains in genus level, *Streptomyces* strains accounted for over half (37 strains). It is quite reasonable, since *Streptomyces* are a well-established source of diverse bioactive compounds possessing antimicrobial activity in the microbial world discovered (Masand et al., 2018; Olanrewaju and Babalola, 2019; Pham et al., 2019). *Nocardiopsis* and *Micromonospora* are the second prevalent genera in the active strains, and each with three strains displayed antibacterial activities. It is noteworthy that two *Nocardiopsis* strains including 14Sc5-11 and 15S9-2, showed less than 98.65% 16S rRNA similarities with the validly reported species in genus *Nocardiopsis*, therefore they might represent two strains in novel species of the genus *Nocardiopsis*. Meanwhile, *Nocardiopsis* species were reported to produce a wide variety of chemical classes of compounds with diverse pharmacological and biological activities (Bennur et al., 2016; Ibrahim et al., 2018). In this context, it would be interesting to explore if novel bioactive metabolites can be identified from the two potential new *Nocardiopsis* strains. Furthermore, five potential new species, including *Cellulosimicrobium* sp. 14Sb3-13 and 14Sb1-5, *Kineococcus* sp. 13S2-4, *Cellulomonas* sp. 10Sc3-5, and *Streptomyces* sp. 20Sb6-6 also showed inhibitory activities against tested pathogens (Supplementary Table S5). Thorough investigations will be carried out further to deepen our knowledge on the antibacterial substances of these potential novel strains.

The action of antimicrobial agents can be generally categorized in five mechanisms: inhibition of cell wall synthesis, alteration of cell membranes structure, inhibition of protein synthesis, inhibition of nucleic acid synthesis, and disruption of the metabolic pathways (such as folic acid and mycolic acid; Wilson, 2014; Kapoor et al., 2017; O’Rourke et al., 2020). In this study, a unique high-throughput model based on a double fluorescent protein reporter “pDualrep2” system was implemented to gain preliminary insights into the mechanism of antibacterial action of bioactive strains. This reporter system can early identify the antibacterial inhibitors targeting three mechanisms in “one-pot” format: DNA damage (expression of rfp reporter), inhibition of protein translation (expression of katushka2S reporter), and others (inhibition of bacterial growth targeting on neither DNA replication nor protein synthesis; Osterman et al., 2016; Ivanenkov et al., 2019a). Sensitivity of this system was highly increased by applying an *E. coli* strain with lacking the *tolC* gene that coded for an essential component of several efflux systems in outer membrane (Zgurskaya et al., 2011; Osterman et al., 2016). Both these effects are clearly observed within the sublethal concentration of an antibacterial sample where it does not kill bacteria but substantially attenuates translation or trigger the SOS response. As a biosensor of antimicrobial activity, the described assay was reported to be successfully applied for identification of a series of promising antibiotics whose antibacterial activity had not been reported previously, such as 2-Guanidino-quinazolines (Komarova et al., 2017), N-pyridyl-substituted carboxypiperidine amides (Ivanenkov et al., 2019b), and N-substituted triazolo-azetidines (Ivanenkov et al., 2019c). Furthermore, in our previous study, this platform contributed to effective discovery of some novel antibiotics that act *via* the translation inhibition mechanism, such as Beilunmycin (Jiang et al., 2020), Hetiamacin E and F (Wang et al., 2020b), acetyl-griseoviridin, and desulphurizing griseoviridin (Wang et al., 2020a). In the present research, two *Streptomyces* strains and two *Saccharothrix* strains induced Katushka2S expression, demonstrating the protein inhibitory activities in their ethyl acetate extractions. Three *Streptomyces* strains demonstrated their inhibition activity against DNA biosynthesis by inducing the RFP expression. Investigation of these strains could be prioritized to find potential antibacterial compounds.

It is generally accepted that rare actinobacterial genera possess more potential in terms of secondary metabolites novelty (Schorn et al., 2016; Bundale et al., 2019; Ding et al., 2019). Thus, exploration of bioactive chemicals from rare actinobacterial strains will be more promising and efficient. In the present study, a *Saccharothrix* strain was analyzed firstly to gain deeper insights into its bioactive metabolites. At the time of writing, the genus *Saccharothrix* encompasses 21 species and 2 subspecies with validly published names, in which eight species were isolated from deserts (Liu et al., 2020c). The genus *Saccharothrix* was found to be a potential producer of novel specialized metabolites, and these chemical entities exhibited a broad range of biological functions, such as antibacterial (Takahashi et al., 1986; Isshiki et al., 1989; Takeuchi et al., 1992), antifungal (Igarashi et al., 1997), antitumor (Vértesy et al., 2001), antivirus (Tomita et al., 1991), pro-inflammatory (Gibson et al., 2005), and other activities (Kimura et al., 1995; Yoshimura et al., 1995; Koguchi et al., 2000) of medical and industrial importance. For instance, at least 14 dithiolopyrrolone compounds with strong activities against a panel of bacteria, yeasts, and filamentous fungi were isolated naturally or using biosynthetic method from *Saccharothrix algeriensis* NRRL B-24137 (Lamari et al., 2002; Bouras et al., 2008; Merrouche et al., 2010, 2011). More than 20 macrolides were isolate from different *Saccharothrix* sp., including cytotoxic 10/11-membered macrolides saccharothriolides A–K, X (Lu et al., 2015, 2016) and 20-membered macrolides ammocidins A–D (Murakami et al., 2001, 2009); antibacterial 16-membered macrolides Tianchimycins A and B (Wang et al., 2013), aldgamycins G, H, and swalpamycin B (Mizobuchi et al., 1986; Zitouni et al., 2007; Wang et al., 2013); and antifungal 16-membered macrolides formamicin (Igarashi et al., 1997). Recently, new antibacterial congeners cyanogriside J and M were isolated from strain ABH26, which was closely related to *S. xinjiangensis* NBRC 101911^T^ (Lahoum et al., 2019); and new cytotoxic compounds, caerulomycin M, saccharopyrone and saccharonoic acid were isolated from *S. xinjiangensis* Act24Zk (Skellam et al., 2013). These promising results emphasize the need to continue the research in the genus *Saccharothrix*. In this research, *Saccharothrix* strain 16Sb2-4 presented a promising antimicrobial potential against all the tested “ESKAPE” pathogens and demonstrated its antibacterial mechanism of interfering with protein translation, thus was given priority for metabolites exploration to discover bioactive compounds from the Taklamakan desert.

To gain a comprehensive understanding of chemical diversity in the cultural broth of strain 16Sb2-4, UPLC-QTof-MS/MS-based dereplication analyses coupled with molecular networking were performed prior to the metabolites isolation. Many researches have reported the successful application of molecular networking for effective chemical dereplication and novel metabolite discovery (Duncan et al., 2015; Kleigrewe et al., 2015; Esposito et al., 2017; Nothias et al., 2018; Lu et al., 2019), especially in our latest research on endophytic actinobacteria from psammophytes in Taklamakan desert, it has successfully contributed to the discovery of two new griseoviridin-type antibiotics from the fermentation broth of an endophytic *Streptomyces* strain (Wang et al., 2020a). In the present study, four families of bioactive compounds were observed and predicted from the crude extract of strain 16Sb2-4, including lipopeptides, cyclic depsipeptides, 22-membered macrolactams, and 16-membered macrolides. The prediction of 16-membered macrolides was further confirmed by efficient isolation and structure elucidation of compounds **8–11**, which evidenced that molecular networking can accelerate dereplication and compound identification, and moreover, can rationalize isolation procedure for targeted purification of bioactive natural products.

Four known 16-membered macrolides, aldgamycin G, aldgamycin H, aldgamycin K, and swalpamycin B, were isolated and identified from *Saccharothrix* sp. 16Sb2-4 by subsequent targeted separation. Meanwhile, all of them exerted the bacteriostatic effect by interfering with protein translation in the pDualrep2 reporter system. These four compounds are all assigned to be 16-membered macrolides containing a rare branched octose unit at C-5 of the macrolactone ring. Many macrolides were reported to often have a bacteriostatic effect on susceptible organisms, caused by inhibition of RNA-dependent protein synthesis through binding to the 50S subunit of the bacterial ribosome (Bolhuis et al., 2011). The sugar substituent at C-5 plays an essential role in their antibacterial activity by direct interaction with 23S RNA (Bolhuis et al., 2011; Karpinski, 2019). It has been reported that aldgamycin G, H and swalpamycin B showed antibacterial activity against Gram-positive bacteria (Mizobuchi et al., 1986; Zitouni et al., 2007; Wang et al., 2013). Although aldgamycin K did not exhibit antimicrobial activity against tested bacteria and fungi in previous report (Wang et al., 2016b), it revealed its antibacterial potential by inhibiting protein synthesis in the highly-sensitive pDualrep2 system in our study. In terms of the biological source, aldgamycin G, aldgamycin H, and swalpamycin B were ever isolated from *Saccharothrix* species before, meanwhile aldgamycin G and swalpamycin B have also been isolated from *Streptomyces* strains (Mizobuchi et al., 1986; Chatterjee et al., 1987). This is the first report of production of aldgamycin K by the genus *Saccharothrix*, which was reported to be produced only by *Streptomyces* strains in previous study (Wang et al., 2016b).

## Conclusion

The study reported a relatively integrated investigation on the diversity, novelty, and pharmacological potential of actinobacterial strains isolated from eight soil samples at various sites along the Alar-Hotan desert highway in the Taklamakan desert. These actinobacterial strains were affiliated to 55 genera in 27 families of 12 orders, which implied significant actinobacterial diversity inhabited in the harsh environment. Over 40% of the 146 tested isolates exhibited bioactive potential against at least one of the tested “ESKAPE” pathogens, among which seven strains demonstrated their ability either to block translation machinery or induce SOS-response in the pDualrep2 system. One strain, *Saccharothrix* sp. 16Sb2-4 as an example was studied to prove the capability to produce diverse secondary metabolites based on in-depth chemical profiling, and four bioactive macrolide antibiotics were purified as secondary metabolites from ISP 2 cultural medium. Exploration of the other bioactive isolates by combination of different strategies, including high-throughput screening, small scale fermentation based on deep-plates, One Strain Many Compounds (OSMAC), and compound dereplication by UPLC-QToF-MS/MS, *etc*., is the subject of our further investigation. It is believed that new antibiotics will be discovered from these desert-derived actinobacterial strains, which will support the idea that the Taklamakan desert represents an undeveloped reservoir of pharmaceutical actinobacteria with possessing a significant capacity to produce novel metabolites with unique antibacterial activity.

## Data Availability Statement

The datasets presented in this study can be found in online repositories. The names of the repository/repositories and accession number(s) can be found in the article/Supplementary Material.

## Author Contributions

CS conceived the whole study, supervised the project, and helped in preparing the manuscript. SL and TW carried out the experiments and prepared the manuscript. CS, FL, and XH contributed in sampling from deserts. QL, GW, ZJ, LL, and XZ helped to prepare some experiments. DL, IO, PS, and OD were responsible for screening the mechanisms of action for samples by means of a double fluorescent protein reporter system. All authors contributed to the article and approved the submitted version.

### Conflict of Interest

The authors declare that the research was conducted in the absence of any commercial or financial relationships that could be construed as a potential conflict of interest.

## Footnotes

^1^http://www.ncbi.nlm.nih.gov/
