# Supplementary material for: Bioprospecting of Soil-Derived Actinobacteria Along the Alar-Hotan Desert Highway in the Taklamakan Desert
Source: Front Microbiol. 2021 Mar 15;12:604999. doi: 10.3389/fmicb.2021.604999 (PMC8005632; doi:10.3389/fmicb.2021.604999)
Supplement: Supplementary file 1 [file Data_Sheet_1.pdf]

Supplemental Material

Bioprospecting of Soil-Derived Actinobacteria along the Alar-Hotan Desert Highway in the Taklamakan Desert

Shao-wei Liu[1,2#], Ting Wang[1#], Qin-pei Lu[1], Fei-na Li[1], Gang Wu[1], Zhong-ke Jiang[1], Xugela Habden[3], Lin Liu[4], Xiao-lin Zhang[5], Dmitrii A. Lukianov[6], Ilya A. Osterman[6,7], Petr V. Sergiev[6,7], Olga A. Dontsova[6,7,8] and Cheng-hang Sun[1,2*]

Table S1. Information of soil samples collected in Taklamakan Desert.

Sample No.	Site information	Altitude/m
S1	40°26'15.81" N, 81°16'23.99" E	1016
S2	40°23'14.96" N, 81°12'24.81" E	1019
S3	40°21'04.32" N, 81°07'25.99" E	1027
S4	40°11'24.37" N, 81°01'12.29" E	1043
S5	39°58'24.83" N, 80°58'33.37" E	1059
S6	39°30'00.67" N, 80°58'58.81" E	1095
S7	38°41'39.16" N, 80°58'46.28" E	1157
S8	38°05'15.88" N, 80°36'36.72" E	1214

Table S2. Compositions of the ten different media used for the isolation of actinobacteria in the study.

NO.	Name	Composition (In 1.0 L distilled water)
M1	Modified Gauze's NO. 1 synthetic medium	Starch 2.0 g, KNO_3 0.5 g, KCl 1.7 g, $MgSO_4$•$7H_2O$ 0.5 g, Na_2HPO_4 0.5 g, NaCl 0.5g, $CaCO_3$ 0.02 g, $FeSO_4$•$7H_2O$ 0.01 g, Vitamin mixture 1.0 mL, Agar 20.0 g, pH 8.0
M2	ISP 2 medium	Yeast extract 4.0 g, Glucose 4.0 g, Malt extract 5.0 g, Vitamin mixture 1.0 mL, Trace salt 1.0 mL, Agar 20.0 g, pH 8.0
M3	R2A medium	R2A (BD) 18.6 g, Agar 12.0 g, pH 8.0
M4	Modified Cellulose-Casein medium	Cellulose 10.0 g, Casein 0.3 g, K_2HPO_4 0.2 g, $FeSO_4$•$7H_2O$ 0.01 g, $CaCO_3$ 0.02 g, KNO_3 2.0 g, $MgSO_4$•$7H_2O$ 0.05 g, NaCl 10g, Agar 20.0 g, pH 8.0
M5	CMKA medium	Casein acids hydrolysate 0.5 g, Mannitol 1.5g, KNO3 1.0 g, $(NH_4)_2SO4$ 2.0 g, K_2HPO_4 0.5g, $CaCO_3$ 0.5g, NaCl 10.0 g, KCl 5.0g, $MgCl_2$ 1.0 g, Agar 20.0 g, pH 8.0
M6	Raffinose-Histidine medium	Raffinose 1.0 g, Histidine 0.1 g, Na_2HPO_4 0.5 g, KCl 1.7g, $MgSO_4$•$7H_2O$ 0.05 g, $FeSO_4$•$7H_2O$ 0.1 g, $CaCO_3$ 0.02 g, Vitamin mixture 1.0 mL, Agar 20.0 g, pH 8.0
M7	Trehalose-Proline medium	Trehalose 5.0 g, L-Proline 1.0 g, $(NH4)_2SO_4$ 1.0 g, $CaCl_2$ 2.0 g, NaCl 1.0 g, K_2HPO_4 1.0 g, $MgSO_4$•$7H_2O$ 1.0 g, Vitamin mixture 1.0 mL, Agar 20.0 g, pH 8.0
M8	Proline medium	L-Proline 5.0 g, Agar 20.0 g, Distilled water 1.0 L, pH 8.0

M9	Casein-Glucose medium	Casein 0.3 g, Glucose 10.0 g, KNO_3 2.0g, $MgSO_4$•$7H_2O$ 0.05 g, K_2HPO_4 2.0 g, $CaCl_2$ 1.0 g, $FeSO_4$•$7H_2O$ 0.01 g, NaCl 50.0 g, KCl 20.0 g, $MgCl_2$•$6H_2O$ 10.0 g, Agar 20.0 g, pH 8.0
M10	Casein-Glucose medium with 16% (w/v) multi-salts	Casein 0.3 g, Glucose 10.0 g, KNO_3 2.0g, $MgSO_4$•$7H_2O$ 0.05 g, K_2HPO_4 2.0 g, $CaCl_2$ 1.0 g, $FeSO_4$•$7H_2O$ 0.01 g, NaCl 100.0 g, KCl 40.0 g, $MgCl_2$•$6H_2O$ 20.0 g, Agar 20.0 g, pH 8.0

Note: Trace salt solution: FeSO4·7H2O 0.2 g, MnCl2.4H2O 0.01 g, ZnSO4·7H2O 0.01 g, distilled water 100.0 ml. Vitamin mixture: thiamine 0.1 g, pyridoxine 0.1 g, riboflavin 0.1g, niacin 0.1 g, biotin 0.1 g, distilled water 100.0 mL.

Table S3. taxonomic statistics of the 590 actinobacterial strains.

Taxon			No. of isolates
**Order (12)**	**Family (27)**	**Genus (55)**	
Propionibacteriales	*Nocardioidaceae*	*Aeromicrobium*	38
		Nocardioides	1
	Kribbellaceae	*Kribbella*	1
	Propionibacteriaceae	*Desertihabitans*	1
Micromonosporales	*Micromonosporaceae*	*Micromonospora*	23
Pseudonocardiales	*Pseudonocardiaceae*	*Saccharothrix*	4
		Amycolatopsis	2
		Saccharopolyspora	1
		Pseudonocardia	2
Streptomycetales	*Streptomycetaceae*	*Streptomyces*	157
Mycobacteriales	*Gordoniaceae*	*Gordonia*	1
	Dietziaceae	*Dietzia*	1
	Nocardiaceae	*Rhodococcus*	1
	Mycobacteriaceae	*Mycolicibacterium*	3
Jiangellales	*Jiangellaceae*	*Jiangella*	2
Streptosporangiales	*Nocardiopsaceae*	*Nocardiopsis*	26
	Streptosporangiaceae	*Nonomuraea*	1
		Streptosporangium	1
	Thermomonosporaceae	*Actinomadura*	1
Micrococcales	*Microbacteriaceae*	*Microbacterium*	95
		Agrococcus	15
		Curtobacterium	3
		Rathayibacter	3
		Pseudolysinimonas	4
		Leucobacter	2
		Pseudoclavibacter	1
		Agromyces	11
		Planctomonas	1
		Salinibacterium	8

		Labedella	16
	Micrococcaceae	*Kocuria*	29
		Micrococcus	6
		Arthrobacter	19
		Zhihengliuella	2
		Neomicrococcus	1
		Rothia	1
		Pseudarthrobacter	2
	Dermabacteraceae	*Brachybacterium*	26
	Promicromonosporaceae	*Cellulosimicrobium*	14
		Isoptericola	4
		Promicromonospora	2
		Oerskovia	2
	Cellulomonadaceae	*Cellulomonas*	8
		Actinotalea	1
	Brevibacteriaceae	*Brevibacterium*	9
	Bogoriellaceae	*Georgenia*	2
	Intrasporangiaceae	*Janibacter*	1
		Ornithinicoccus	1
	Ruaniaceae	*Haloactinobacterium*	1
Kineosporiales	*Kineosporiaceae*	*Kineococcus*	16
Geodermatophilales	*Geodermatophilaceae*	*Blastococcus*	12
		Geodermatophilus	1
		Modestobacter	1
Nakamurellales	*Nakamurellaceae*	*Nakamurella*	1
Solirubrobacterales	*Patulibacteraceae*	*Patulibacter*	2
Total number of actinobacterial isolates			590

Table S4. Taxonomic classification of 33 potential new species and 4 published new species (bold fonts) based on BLAST results of their nearly full-length 16S rRNA gene sequence (>1300 bp) in EzBiocloud database. Among the 37 strains, 36 strains (NO. 1−36) exhibited less than 98.65% similarities to the top BLAST hits.

NO.	Strain NO.	GenBank Accession NO.	Length of blasted 16S rRNA gene sequences (bp)	Top-hit taxon	Similarity (%)	Family assignment
1	**13S1-3**[a]	**MH287062**	**1481**	***Frondihabitans australicus* DSM 17894**^{T}	**97.29**	***Microbacteriaceae***
2	21Sc5-12	MT682463	1384	*Salinibacterium hongtaonis* 194^{T}	97.76	*Microbacteriaceae*
3	15S1-1	MT682465	1380	*Agromyces arachidis* AK-1^{T}	97.95	*Microbacteriaceae*
4	15S6-12	MT682451	1487	*Salinibacterium hongtaonis* 194^{T}	97.99	*Microbacteriaceae*
5	21Sb5-5	MT682467	1372	*Salinibacterium hongtaonis* 194^{T}	98.03	*Microbacteriaceae*
6	20Sb3-5	MT682429	1484	*Pseudolysinimonas kribbensis* MSL-13^{T}	98.13	*Microbacteriaceae*
7	21Sb2-13	MT682471	1354	*Salinibacterium hongtaonis* 194^{T}	98.15	*Microbacteriaceae*
8	16Sc1-5	MT682470	1484	*Pseudolysinimonas kribbensis* MSL-13^{T}	98.16	*Microbacteriaceae*
9	20Sb5-7	MT682472	1361	*Microbacterium wangchenii* dk512^{T}	98.16	*Microbacteriaceae*
10	16Sc5-2	MT682476	1348	*Microbacterium wangchenii* dk512^{T}	98.44	*Microbacteriaceae*
11	14Sc6-4	MT682478	1387	*Brachybacterium sacelli* LMG 20345^{T}	98.37	*Dermabacteraceae*
12	10Sb10-5	MT682479	1382	*Brachybacterium sacelli* LMG 20345^{T}	98.37	*Dermabacteraceae*
13	14Sb5-3	MT682483	1374	*Brachybacterium sacelli* LMG 20345^{T}	98.43	*Dermabacteraceae*
14	12S10-3	MT682484	1378	*Brachybacterium sacelli* LMG 20345^{T}	98.44	*Dermabacteraceae*
15	14Sc6-3	MT682487	1361	*Brachybacterium squillarum* M-6-3^{T}	98.49	*Dermabacteraceae*

16	14Sb1-5	MT682466	1384	*Cellulosimicrobium cellulans* LMG 16121^{T}	97.97	*Promicromonosporaceae*
17	14Sb8-3	MT682468	1379	*Cellulosimicrobium cellulans* LMG 16121^{T}	98.04	*Promicromonosporaceae*
18	14Sb3-13	MT682474	1354	*Cellulosimicrobium cellulans* LMG 16121^{T}	98.23	*Promicromonosporaceae*
19	14Sb1-13	MT682402	1351	*Actinotalea ferrariae* CF5-4^{T}	98.30	*Cellulomonadaceae*
20	10Sc3-5	MT682477	1323	*Cellulomonas aerilata* 5420S-23^{T}	98.34	*Cellulomonadaceae*
21	10Sb1-2	MT682485	1360	*Cellulomonas aerilata* 5420S-23^{T}	98.45	*Cellulomonadaceae*
22	**21Sc5-5**[b]	**MK787305**	**1480**	***Nocardioides albidus* THG-S11.7**^{T}	**97.30**	***Nocardioidaceae***
23	21Sa5-10	MT682480	1360	*Aeromicrobium marinum* DSM 15272^{T}	98.38	*Nocardioidaceae*
24	14Sb8-8	MT682481	1303	*Aeromicrobium marinum* DSM 15272^{T}	98.62	*Nocardioidaceae*
25	14Sb6-5	MT682473	1390	*Nocardiopsis halotolerans* DSM 44410^{T}	98.20	*Nocardiopsaceae*
26	15S9-2	MT682482	1381	*Nocardiopsis halotolerans* DSM 44410^{T}	98.41	*Nocardiopsaceae*
27	14Sc5-11	MT682486	1363	*Nocardiopsis halotolerans* DSM 44410T	98.46	*Nocardiopsaceae*
28	20Sb1-6	MT682462	1345	*Streptomyces tsukubensis* NRRL18488^{T}	97.55	*Streptomycetaceae*
29	20Sb6-6	MT682490	1359	*Streptomyces tyrosinilyticus* NEAU-Jh-20^{T}	97.86	*Streptomycetaceae*
30	13S5-5	MT682475	1371	*Streptomyces salilacus* TRM 41337^{T}	98.25	*Streptomycetaceae*
31	13S2-4	MT682489	1324	*Kineococcus endophyticus* KLBMP 1274^{T}	98.64	*Kineosporiaceae*
32	10S2-1	MT682424	1336	*Kineococcus endophyticus* KLBMP 1274^{T}	98.65	*Kineosporiaceae*
33	10Sc9-8	MT682461	1493	*Georgenia muralis* DSM 14418^{T}	97.16	*Bogoriellaceae*
34	13Sb5-2	MT682417	1482	*Geodermatophilus obscurus* DSM 43160^{T}	98.06	*Geodermatophilaceae*
35	**12Sc4-1**[c]	**MH244160**	**1478**	***Nakamurella silvestris* S20-107**^{T}	**96.94**	***Nakamurellaceae***

36	12S1-3	MT682469	1494	*Patulibacter minatonensis* DSM 18081^T	97.94	*Patulibacteraceae*
**37**	**16Sb5-5[d]**	**MK947033**	**1478**	***Desertihabitans aurantiacus* CPCC 204711^T**	**99.60**	***Propionibacteriaceae***

Note:

[a] Published as *Planctomonas deserti* gen. nov., sp. nov., a novel species of a new genus in the family *Microbacteriaceae*. [Liu, S.W., Li, F.N., Zheng, H.Y., Qi, X., Huang, D.L., and Xie, Y.Y., et al. (2019a). *Planctomonas deserti* gen. nov., sp. nov., a new member of the family *Microbacteriaceae* isolated from soil of the Taklamakan desert. *Int. J. Syst. Evol. Microbiol*. 69, 616-624. doi:10.1099/ijsem.0.003095].

[b] Published as *Nocardioides vastitatis* sp. nov., a novel species of the genus *Nocardioides*. [Liu, S.W., Xue, C.M., Li, F.N., and Sun, C.H. (2020a). *Nocardioides vastitatis* sp. nov., isolated from Taklamakan desert soil. *Int. J. Syst. Evol. Microbiol*. 70, 77-82. doi:10.1099/ijsem.0.003718].

[c] Published as *Nakamurella deserti* sp. nov., a novel species of the genus *Nakamurella*. [Liu, S.W., Li, F.N., Qi, X., Xie, Y.Y., and Sun, C.H. (2019b). *Nakamurella deserti* sp. nov., isolated from rhizosphere soil of *Reaumuria* in the Taklamakan desert. *Int. J. Syst. Evol. Microbiol*. 69, 214-219. doi:10.1099/ijsem.0.003132].

[d] Published as *Desertihabitans brevis* sp. nov., a novel species of the genus *Desertihabitans*. [Liu, S.W., Li, F.N., Liu, H.Y., Yu, L.Y., and Sun, C.H. (2020b). *Desertihabitans brevis* sp. nov., an actinobacterium isolated from sand of the Taklamakan desert, and emended description of the genus *Desertihabitans*. *Int. J. Syst. Evol. Microbiol*. 70, 1166-1171. doi:10.1099/ijsem.0.003896].

Table S5. Antibacterial activity of 61 active actinobacterial strains isolated from soil samples collected in Taklamakan desert.

NO.	Isolates (GenBank accession NO.)	Top-hit taxon (Pairwise similarity)	Fractions[a]	Activity[b]											
				E. coli		*P. aeruginosa*		*K. peneumoniae*		*A. baumannii*		*S. aureus*		*E. faecalis*	
				S	R	S	R	S	R	S	R	S	R	S	R
1	14Sc5-7 (MT705174)	*Streptomyces abyssomicinicus* CHI39^T, 99.87%	E	—	—	—	—	—	—	—	—	—	—	—	—
			W	—	—	—	—	—	—	—	—	0.69	—	—	—
			M	—	—	—	—	—	—	—	—	—	—	—	—
2	13S1-2 (MT682452)	*Streptomyces amritsarensis* MTCC 11845^T, 100%	E	—	—	—	—	—	—	0.72	—	1.06	1.10	1.02	0.92
			W	—	—	—	—	—	—	—	—	0.82	1.05	—	—
			M	—	—	—	—	—	—	—	—	0.90	—	—	—
3	15S3-3 (MT705170)	*Streptomyces atroolivaceus* NRRL ISP-5137^T, 99.68%	E	—	—	—	—	—	—	—	—	—	—	—	0.78
			W	—	—	—	—	—	—	—	—	—	—	—	—
			M	—	—	—	—	—	—	—	—	—	—	—	—
4	10S9-4 (MT705189)	*Streptomyces atrovirens* NRRL B-16357^T, 99.26%	E	1.13	1.11	—	—	1.31	1.18	1.10	1.21	1.26	1.42	—	—
			W	—	—	—	—	—	—	—	—	—	—	—	—
			M	—	—	—	—	—	—	—	—	—	—	—	—

5	16S9-2 (MT705190)	*Streptomyces atrovirens* NRRL B-16357^{T}, 99.58%	E	—	—	—	—	—	—	—	—	0.97	1.00	0.92	—
			W	—	—	—	—	—	—	—	—	—	—	—	—
			M	—	—	—	—	—	—	—	—	0.65	—	—	—
6	13S9-1 (MT705175)	*Streptomyces badius* NRRL B-2567^{T}, 99.90%	E	1.40	1.54	1.68	1.43	1.01	1.25	1.50	1.59	1.62	1.60	1.31	—
			W	—	—	—	—	0.67	—	—	—	—	—	—	—
			M	—	—	—	—	—	—	—	—	—	—	—	—
7	21Sc2-8 (MT705200)	*Streptomyces caeruleatus* NRRL B-24802^{T}, 99.89%	E	—	—	—	—	—	—	—	—	—	—	—	—
			W	—	—	—	—	—	—	—	—	0.71	0.87	—	—
			M	—	—	—	—	—	—	—	—	—	—	—	—
8	12S8-3 (MT705184)	*Streptomyces coelescens* DSM 40421^{T}, 100%	E	—	—	—	—	—	—	—	—	—	—	—	—
			W	1.28	0.70	—	—	1.10	—	—	—	—	—	—	—
			M	—	—	—	—	—	—	—	—	—	—	—	—
9	12S1-1 (MT705171)	*Streptomyces coeruleoprunus* NBRC 15400^{T}, 99.74%	E			—	—	1.23	—	1.62	1.69	1.05	0.89	—	—
			W	—	—	—	—	—	—	—	—	—	—	—	—
			M	—	—	1.44	—	—	—	—	—	—	—	—	—
10	14Sb3-7 (MT705177)	*Streptomyces diacarni* LHW51701^{T}, 99.34%	E		—	—	—	0.78	—	0.90	1.36	0.97	1.23	1.69	2.25
			W	—	—	—	—	—	—	—	—	—	0.73	—	—

			M	—	—	—	—	—	—	—	—	0.64	0.73	1.18	—
11	12S8-2	*Streptomyces dioscori* A217^T,	E	—	—	—	—		—	—	—	1.50	1.40	1.18	1.04
	(MT705181)	99.37%	W	—	—	—	—	—	—	—	—	1.09	1.71	1.51	1.25
			M	—	—	—	—	—	—	—	—	1.17	1.11	1.02	1.11
12	14Sb1-17	*Streptomyces erythrogriseus* LMG	E	0.65	—	—	—	0.72	—	—	—	1.15	1.15	0.70	1.30
	(MT705193)	19406^T, 100%	W	1.28	0.94	—	—	1.11	0.88	—	0.90	1.38	1.40	—	1.40
			M	—	—	—	—	—	—	—	—	—	—	—	—
13	12S8-1	*Streptomyces flavoviridis* NBRC	E	—	—	—	—	1.09	—	—	—	1.21	1.14	1.00	1.25
	(MT705173)	12772^T, 99.07%	W	—	—	—	—	—	—	—	—	—	—	—	—
			M	—	—	—	—	—	—	—	—	1.00	0.70	—	—
14	15S5-12	*Streptomyces flavoviridis* NBRC	E	—	—	—	—	—	—	—	—	1.00	0.94	—	—
	(MT705188)	12772^T, 99.23%	W	—	—	—	—	—	—	—	—	—	—	—	—
			M	—	—	—	—	—	—	—	—	—	—	—	—
15	13S2-11	*Streptomyces glomeroaurantiacus*	E		0.91	—	—	0.87	—	—	—	2.02	1.70	1.30	2.20
	(MT705187)	NBRC 15418^T, 99.38%	W	—	—	—	—	—	—	—	—	—	—	—	—
			M	—	—	—	—	—	—	—	—	0.90	0.68	—	0.87
16	20S9-6	*Streptomyces griseoincarnatus*	E	—	—	—	—	0.91	—	—	—	1.20	1.62	1.60	1.18

	(MT705196)	LMG 19316^T, 100%	W	0.85	—	—	—	0.90	0.83	1.28	1.65	1.50	1.42	1.08	1.22
			M	—	—	—	—	—	—	—	—	2.05	1.12	—	—
17	15S3-2	*Streptomyces griseoviridis* NBRC	E	—	—	—	—	—	—	—	—	—	—	0.70	0.75
	(MT705182)	12874^T, 98.85%	W	—	—	—	—	—	—	—	—	—	—	—	—
			M	—	—	—	—	—	—	—	—	—	—	—	—
18	14Sb4-3	*Streptomyces griseus* subsp. *griseus*	E	1.50	1.12	0.92	0.94	1.61	0.72	0.77	1.00	1.72	1.73	1.28	2.25
	(MT705168)	KCTC 9080^T, 99.68%	W	1.14	—	—	—	1.40	—	—	—	—	—	—	—
			M	—	—	—	—	0.84	—	—	—	—	—	—	—
19	12S6-2	*Streptomyces halstedii* NBRC	E	—	—	—	—	—	—	—	—	0.83	0.89	1.05	1.24
	(MT705169)	12783^T, 100%	W	—	—	—	—	—	—	—	—	—	—	—	—
			M	—	—	—	—	0.96	—	—	—	1.10	1.05	—	—
20	13S9-4	*Streptomyces heliomycini* NBRC	E	—	—	—	—	—	—	—	—	—	1.05	—	—
	(MT705185)	15899^T, 99.75%	W	—	—	—	—	—	—	—	—	—	—	—	—
			M	—	—	—	—	—	—	—	—	—	—	—	—
21	15S7-1	*Streptomyces indoligenes* TRM	E	—	—	—	—	—	—	—	—	0.75	0.79	0.80	0.72
	(MT705192)	43006T, 99.68%	W	—	—	—	—	—	—	—	—	—	—	—	—
			M	—	—	—	—	—	—	—	—	—	—	—	—

22	20Sa2-4 (MT705197)	*Streptomyces luozhongensis* TRM 49605^T, 99.16%	E	—	—	—	—	—	—	1.40	—	1.70	—	—	—
			W	—	—	—	—	—	—	—	—	—	—	—	—
			M	—	—	—	—	—	—	—	—	—	—	—	—
23	14Sb8-6 (MT705191)	*Streptomyces luridiscabiei* NRRL B-24455^T, 100%	E	0.68	0.67	—	—			—	0.70	—	—	—	—
			W	—	—	—	—	—	—	—	—	—	—	—	—
			M	—	—	—	—	—	—	—	—	—	—	—	—
24	21Sa10-2 (MT705202)	*Streptomyces luteus* TRM 45540^T, 99.67%	E	—	0.75	—	—	—	—	—	—	1.45	1.50	1.71	1.15
			W	—	—	—	—	—	—	—	—	—	0.75	—	—
			M	—	—	—	—	—	—	—	—	—	—	—	—
25	21Sb1-24 (MT705203)	*Streptomyces luteus* TRM 45540^T, 100%	E	—	—	—	—	—	—	—	—	—	—	—	—
			W	—	—	—	—	—	—	—	—	—	—	—	—
			M	—	—	—	—	—	—	—	—	1.30	1.40		1.30
26	21Sa3-10 (MT705201)	*Streptomyces mutabilis* NBRC 12800^T, 100%	E	—	—	—	—	—	—	—	—	1.10	1.20	0.85	1.10
			W	—	—	—	—	—	—	—	—	—	—	—	—
			M	—	—	—	—	—	—	—	—	—	—	—	—
27	13S2-9 (MT705183)	*Streptomyces nogalater* JCM 4799^T, 99.21%	E	—	—	—	—	—	—	—	—	0.93	0.94	0.91	1.41
			W	—	—	—	—	—	—	—	—	—	—	—	—

			M	—	—	—	—	—	—	—	—	—	—	—	—
28	15S4-1 (MT705176)	*Streptomyces omiyaensis* NBRC 13449^T, 98.96%	E	0.94	1.03	—	—	0.80	0.92	—	0.66	0.92	0.84	1.12	—
			W	—	—	—	—	—	—	—	—	—	—	—	—
			M	—	—	—	—	—	—	—	—	—	—	—	—
29	20Sa8-5 (MT705199)	*Streptomyces plicatus* NBRC 13071^T, 100%	E	—	—	—	—	1.40	—	0.75	—	0.85	0.95	1.35	1.85
			W		1.02	—	—	—	0.80	1.19	0.92	—	—	—	—
			M	—	—	—	—	0.98	—	—	—		—	0.90	0.98
30	14Sb3-6 (MT705179)	*Streptomyces pratensis* ch24^T, 100%	E	—	—	—	—	—	—	—	—	0.68	0.66	1.15	1.20
			W	—	—	—	—	—	—	—	—			1.00	1.08
			M	—	—	1.12	—	0.86	—	—	—	0.68	—	1.30	1.35
31	13S1-1 (MT705178)	*Streptomyces puniceus* NRRL ISP-5058^T, 100%	E	—	—	—	—	—	—	—	—	1.02	0.95	0.93	1.14
			W	—	—	—	—	—	—	—	—	1.03	0.91	1.10	1.14
			M	—	—	—	—	—	—	—	—	0.88	0.97	0.90	0.90
32	10Sb5-5 (MT705186)	*Streptomyces qinglanensis* 172205^T, 99.03%	E	1.41	1.18	—	—	1.55	1.20	—	—	1.30	1.55	1.10	1.08
			W	—	—	—	—	—	—	—	—	—	—	—	—
			M	—	—	—	—	—	—	—	—	—	—	—	—
33	15S5-2	*Streptomyces rectiviolaceus* NRRL	E	—	—	—	—	—	—	—	—	1.34	1.32	1.09	1.20

	(MT705172)	B-16374^T, 100%	W	—	—	—	—	0.63	0.70	0.70	—	—	—	—	—
			M	—	—	—	—	—	—	—	—	0.90	1.04	—	—
34	20Sa9-14	*Streptomyces spinoverrucosus*	E	1.00	—	—	—	0.82	—	—	—	1.39	1.86	1.85	2.30
	(MT705204)	NBRC 14228^T, 99.45%	W	—	—	—	—	—	—	—		—	—	—	—
			M	—	—	—	—	—	—	—	—	—	—	—	—
35	16S2-5	*Streptomyces thinghirensis* DSM	E	—	—	—	—	—	—	—	—	0.70	0.71	—	—
	(MT705180)	41919^T, 99.89%	W	—	—	—	—	—	—	—	—	—	—	—	—
			M	—	—	—	—	—	—	—	—	—	—	—	—
36	20Sa3-1	*Streptomyces thinghirensis* DSM	E	—	—	—	—	—	—	—	—	0.80	—	—	—
	(MT705198)	41919^T, 99.62%	W	—	—	—	—	—	—	—	—	—	—	—	—
			M	—	—	—	—	—	—	—	—	1.10	1.00	—	—
37	20Sb6-6	*Streptomyces tyrosinilyticus*	E	—	—	—	—	—	—	—	—	1.10	—	1.10	—
	(MT682490)	NEAU-Jh-20^T, 97.86%	W	—	—	—	—	—	—	—	—	—	—	—	—
			M	—	—	—	—	—	—	—	—	—	—	—	—
38	12Sb1-6	*Nocardiopsis alba* DSM 43377^T,	E	—	—	—	—	—	—	—	—	—	—	—	—
	(MT705166)	99.35%	W	—	—	—	—	—	—	—	—	—	—	—	—
			M	—	—	1.80	—	—	—	—	—	—	—	—	—

39	14Sc5-11	*Nocardiopsis halotolerans* DSM	E	1.00	1.24	1.13	1.03	1.08	1.07	0.75	0.90	1.20	0.91	1.20	2.08
	(MT682486)	44410^{T}, 98.46%	W	0.69	0.85	—	—	0.71	0.69	—	—	—	—	—	1.45
			M	0.79	0.78	0.62	0.72	0.70	0.73	—	—	—	—	—	—
40	15S9-2	*Nocardiopsis halotolerans* DSM	E	1.15	1.00	—	—	1.90	0.90	0.80	1.45	1.90	2.05	1.25	1.25
	(MT682482)	44410^{T}, 98.41%	W	—	—	—	—	—	—	—	—	—	—	—	—
			M	—	—	—	—	—	—	—	—	—	—	—	—
41	12Sd5-1	*Micromonospora andamanensis*	E	—	—	—	—	—	—	—	—	—	—	—	—
	(MT705167)	SP03-05^{T}, 100%	W	—	—	—	—	—	—	—	—	—	—	—	—
			M	—	—	1.91	—	—	—	—	—	—	—	—	—
42	20Sb6-7	*Micromonospora cremea* DSM	E	—	—	—	—	—	—	—	—	0.75	—	—	—
	(MT705194)	45599^{T}, 99.77%	W	—	—	—	—	—	—	—	—	—	—	—	—
			M	—	—	—	—	—	—	—	—	—	—	—	—
43	20Sa3-6	*Micromonospora narathiwatensis*	E	—	—	—	—	—	—	—	—	—	—	—	—
	(MT705195)	DSM 45248^{T}, 99.4%	W	—	—	—	—	—	—	—	—	—	—	—	—
			M	—	—	—	—	—	—	—	—	0.75	—	—	—
44	14Sb3-13	*Cellulosimicrobium cellulans* LMG	E	—	—	—	—	—	—	—	—	0.69		—	—
	(MT682474)	16121^{T}, 98.23%	W	—	—	—	—	—	—	—	—	—	—	—	—

			M	—	—	—	—	—	—	—	—	—	—	—	—
45	14Sb1-5	*Cellulosimicrobium cellulans* LMG	E		1.48	—	—	—	—	—	—	—	—	—	—
	(MT682466)	16121^T, 97.97%	W	—	—	—	—	—	—	—	—	—	—	—	—
			M	—	—	—	—	—	—	—	—	—	—	—	—
46	16Sb5-9	*Microbacterium ginsengisoli* DSM	E	—	—	—	—	—	—	—	—	—	—	—	—
	(MT705161)	18659^T, 98.75%	W	—	—	—	—	—	—	—	—	—	—	—	—
			M	—	—	—	—	—	—	—	—	0.67	0.78	—	—
47	14Sc5-17	*Microbacterium aquimaris* JS54-2	E	1.47	1.31	1.71	1.55	0.96	1.50	1.40	1.63	1.50	1.67	1.38	—
	(MT705163)	^{T}, 99.13%	W	—	—	—	—	1.12	—	—	—	—	—	—	—
			M	—	—	—	—	—	—	—	—	—	—	—	—
48	14Sd3-3	*Saccharothrix longispora* NRRL	E	—	—	—	—	1.15	—	0.78	0.74	1.21	1.20	—	1.75
	(MT682450)	B-116116^T, 100%	W	—	—	—	—	—	—	—	—	—	—	—	—
			M	—	—	—	—	0.68	—	—	—	—	—	—	—
49	16Sb2-4	*Saccharothrix xinjiangensis* NBRC	E	1.81	2.17	1.51	1.68	1.82	1.07	1.66	1.30	2.30	2.28	1.65	2.42
	(MT705162)	101911^T, 99.71%	W	—	—	—	—	1.19	—	0.63	—	1.28	1.05	0.91	1.40
			M	—	—	1.68	—	0.91	—	—	—	1.37	1.20	1.50	0.91
50	21Sc5-16	*Actinomadura apis* IM17-1^T,	E	—	—	—	—	—	—	—	—	—	—	—	—

	(MT682401)	99.17%	W	—	—	—	—	—	—	—	—	0.70	0.79	—	—
			M	—	—	—	—	—	—	—	—	0.65	0.90	—	—
51	15S6-5 (MT682403)	*Aeromicrobium massiliense* JC14^T, 100%	E	—	—	—	—	0.67	—	—	—	—	—	—	—
			W	—	—	—	—	—	—	—	—	—	—	—	—
			M	—	—	—	—	—	—	—	—	—	—	—	—
52	10Sb2-2 (MT705165)	*Blastococcus saxobsidens* BC448 ^{T}, 99.74%	E	—	—	—	—	—	—	—	—	—	—	—	—
			W	—	—	—	—	—	—	—	—	0.65	0.74	—	—
			M	—	—	—	—	—	—	—	—	—	—	—	—
53	10Sc3-5 (MT682477)	*Cellulomonas aerilata* 5420S-23^T, 98.34%	E	—	—	—	—	—	—	—	—	0.65	0.68	—	—
			W	—	—	—	—	—	—	—	—	—	—	—	—
			M	—	—	—	—	—	—	—	—	—	—	—	—
54	16Sb5-5 (MK947033)	*Desertihabitans aurantiacus* CPCC 204711^T, 99.60%	E	—	—	—	—	—	—	—	—	0.75	0.74	—	0.70
			W	—	—	—	—	—	—	—	—	—	—	—	—
			M	—	—	—	—	—	—	—	—	—	—	—	—
55	10S2-3 (MT682422)	*Janibacter melonis* CM2104^T, 99.87%	E	—	—	—	—	—	—	—	—	—	—	—	—
			W	—	—	—	—	—	—	—	—	—	—	—	—
			M	—	—	—	—	—	—	—	—	0.79	0.96	0.88	0.92

56	13S2-4 (MT682489)	*Kineococcus endophyticus* KLBMP 1274^{T}, 98.64%	E	—	—	—	—	—	—	—	—	0.84	0.91	0.98	1.06
			W	—	—	—	—	—	—	—	—	—	—	—	—
			M	—	—	—	—	—	—	—	—	0.91	0.93	0.92	1.03
57	10S3-4 (MT705160)	*Kocuria rosea* DSM 20447^{T}, 99.75%	E	—	—	—	—	—	—	—	—	—	—	—	—
			W	0.68	—	—	—	—	0.67	—	—	—	—	—	—
			M	—	—	—	—	—	—	—	—	0.72	0.76	—	—
58	13S8-7 (MT705164)	*Leucobacter chromiiresistens* JG 31^{T}, 99.50%	E	—	—	—	—	—	—	—	—	0.68	—	—	—
			W	—	—	—	—	—	—	—	—	—	—	—	—
			M	—	—	—	—	—	—	—	—	—	—	—	—
59	13S1-3 (MH287062)	*Planctomonas deserti* 13S1-3^{T}, 100%	E	—	—	—	—	—	—	—	—	—	0.77	—	—
			W	—	—	—	—	—	—	—	—	—	—	—	—
			M	—	—	—	—	—	—	—	—	—	—	—	—
60	16Sc1-5 (MT682470)	*Pseudolysinimonas kribbensis* MSL-13^{T}, 98.16%	E	—	—	—	—	—	—			0.68		—	—
			W	—	—	—	—	—	—	—	—	—	—	—	—
			M	—	—	—	—	—	—	—	—	—	—	—	—
61	21S2-18 (MT682445)	*Pseudonocardia kunmingensis* YIM 63158^{T}, 99.31%	E	—	—	—	—	—	—	—	—	—	—	—	—
			W	—	—	—	—	—	—	—	—	—	—	—	1.05

M	—	—	—	—	—	—	—	—	—	—	—	—

[a] E: crude sample extracted with ethyl acetate; M: crude sample from mycelium; W: crude sample from water layer;

[b] The diameters of the inhibition zones: mm; —, no inhibition.

Table S6. Putatively identified metabolites from liquid culture extracts of *Saccharothrix* sp. 16Sb2-4 based on GNPS database and manual dereplication with UNIFI software by searching the microbial natural products database, The Natural Products Atlas (www.npatlas.org). tR, retention time in UPLC.

Compound No.	tR (Second)	Observed m/z	Calculated m/z	Putative chemical	Protonated adducts	Molecular formula	Chemical structure
1	1550.51	1036.685	1035.683	Surfactin C	$[M+H]^+$	$C_{53}H_{93}N_7O_{13}$	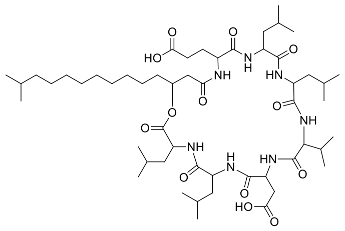
2	1509.32	1022.667	1022.668	Surfactin C14	$[M+H]^+$	$C_{52}H_{91}N_7O_{13}$	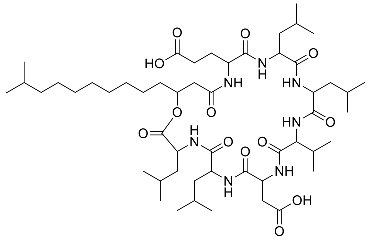

3	1573.49	1022.675	1022.667	[Val7]-Surfactin C15	$[M+H]^{+}$	$C_{52}H_{91}N_7O_{13}$	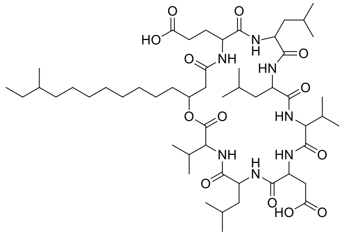
4	813.16	411.295	411.289	Xenotetrapeptide	$[M+H]^{+}$	$C_{21}H_{38}N_4O_4$	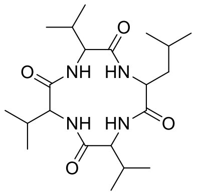
5	1448.11	301.155	301.153	YM-47142	$[M+3H]^{3+}$	$C_{43}H_{64}N_8O_{13}$	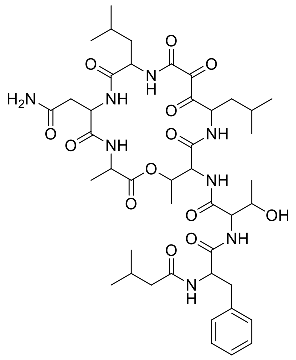
6	623.16	486.286	486.278	Dracolactam A	$[M+H]^{+}$	$C_{28}H_{39}NO_6$	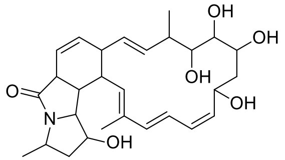

7	720.98	470.290	470.283	Micromonolactam	$[M+H]^+$	$C_{28}H_{39}NO_5$	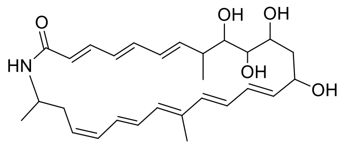
8	819.25	715.392	715.383	Aldgamycin H	$[M+H]^+$	$C_{36}H_{58}O_{14}$	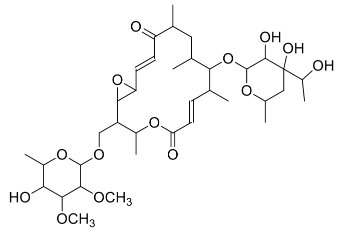
9	778.62	717.407	716.398	Aldgamycin K	$[M+H]^+$	$C_{36}H_{60}O_{14}$	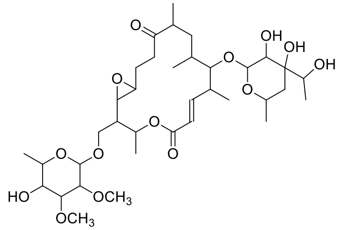
10	977.34	741.368	741.362	Aldgamycin G	$[M+H]^+$	$C_{37}H_{56}O_{15}$	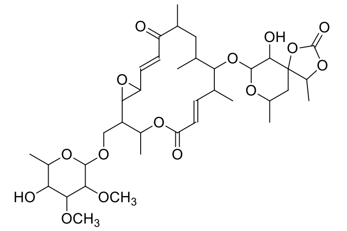

11	821.58	699.452	699.388	Swalpamycin B	$[M+H]^+$	$C_{36}H_{58}O_{13}$	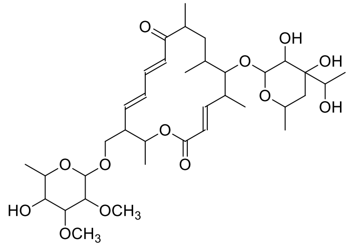
12	896.90	727.353	727.356	Aldgamycin M	$[M+H]^+$	$C_{37}H_{58}O_{14}$	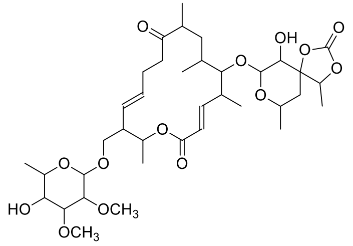
13	925.75	728.385	728.414	Mycinamicin II	$[M+H]^+$	$C_{37}H_{61}NO_{13}$	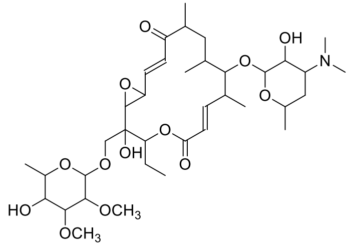

14	791.08	541.303	541.293	5-*O*-dealdgarosyl-aldgamycin G	$[M+H]^+$	$C_{28}H_{44}O_{10}$	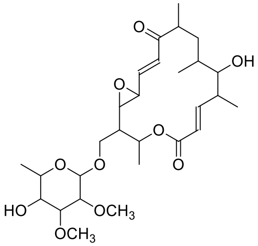
15	949.61	567.280	567.273	14-*O*-demycinosyl-aldgamycin G	$[M+H]^+$	$C_{29}H_{42}O_{11}$	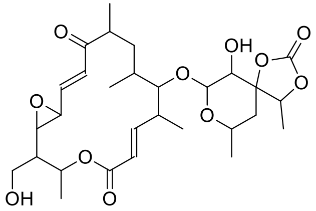
16	832.54	555.317	555.309	5-*O*-dedesosaminyl-mycinamicin V	$[M+H]^+$	$C_{29}H_{46}O_{10}$	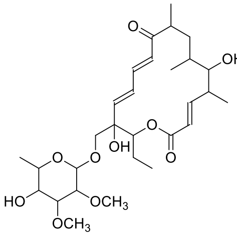

Table S7. NMR data of chemical structures of compounds **8–11**.

^{1}H NMR and ^{13}C NMR data of aldgamycin H (8)

^{1}H NMR (600 MHz, $CDCl_3$) *δ* 6.62 (dd, *J* = 15.4, 10.7 Hz, 1H), 6.56 (d, *J* = 15.4 Hz, 1H), 6.41 (dd, *J* = 15.4, 9.3 Hz, 1H), 5.80 (d, *J* = 15.4 Hz, 1H), 5.34 (dq, *J* = 12.4, 6.2 Hz, 1H), 4.60 (d, *J* = 7.6 Hz, 1H), 4.56 (d, *J* = 7.7 Hz, 1H), 4.16 (dd, *J* = 10.1, 2.9 Hz, 1H), 3.95 – 3.87 (m, 1H), 3.75 (t, *J* = 2.8 Hz, 1H), 3.68 – 3.64 (m, 1H), 3.63 (dd, *J* = 10.3, 3.0 Hz, 1H), 3.60 (s, 3H), 3.60 – 3.58 (m, 1H), 3.54 (s, 3H), 3.52 (dd, *J* = 9.1, 6.3 Hz, 1H), 3.33 (d, *J* = 10.2 Hz, 1H), 3.26 (d, *J* = 9.3 Hz, 1H), 3.21 – 3.16 (m, 1H), 3.09 – 3.05 (m, 2H), 2.83 (s, 1H), 2.74 – 2.66 (m, 1H), 2.62 – 2.55 (m, 1H), 2.37 (s, 1H), 1.63 – 1.53 (m, 2H), 1.53 – 1.47 (m, 1H), 1.37 (dd, *J* = 10.5, 5.7 Hz, 2H), 1.32 (d, *J* = 6.3 Hz, 3H), 1.29 – 1.23 (m, 6H), 1.23 – 1.17 (m, 4H), 1.16 (d, *J* = 6.2 Hz, 3H), 1.12 (d, *J* = 6.9 Hz, 3H), 0.97 (d, *J* = 6.8 Hz, 2H);

^{13}C NMR (150 MHz, $CDCl_3$) *δ* 200.97, 165.42, 151.16, 144.13, 125.63, 120.64, 101.76, 101.02, 86.70, 81.98, 79.73, 74.10, 73.76, 72.79, 72.59, 70.82, 68.79, 67.13, 66.97, 61.80, 59.78, 59.13, 59.00, 49.56, 44.72, 41.87, 39.38, 34.18, 32.10, 20.88, 18.97, 18.51, 18.23, 17.88, 17.72, 17.08.

Note: spectrum recorded obtained in this study matches that reported by Zitounni *et al*. (2007), aldgamycin H.

^{1}H NMR and ^{13}C NMR data of aldgamycin K (9)

1H NMR (600 MHz, CD_3OD) δ 6.79 (dd, J = 15.5, 10.6 Hz, 1H), 5.98 (d, J = 15.5 Hz, 1H), 5.32 (dq, J = 12.5, 6.2 Hz, 1H), 4.61 (d, J = 7.9 Hz, 1H), 4.55 (d, J = 8.0 Hz, 1H), 4.07 (dd, J = 10.0, 3.5 Hz, 1H), 3.92 – 3.84 (m, 2H), 3.77 (t, J = 2.7 Hz, 1H), 3.68 – 3.61 (m, 2H), 3.57 (s, 3H), 3.55 (s, 1H), 3.54 (s, 3H), 3.44 (d, J = 9.8 Hz, 1H), 3.16 (dd, J = 9.6, 2.7 Hz, 1H), 3.06 (dd, J = 8.0, 2.7 Hz, 1H), 2.88 (d, J = 8.2 Hz, 1H), 2.84 – 2.77 (m, 1H), 2.75 – 2.72 (m, 1H), 2.70 (dd, J = 10.6, 5.6 Hz, 1H), 2.59 – 2.50 (m, 1H), 2.14 – 2.05 (m, 1H), 1.97 (s, 1H), 1.84 – 1.76 (m, 1H), 1.56 – 1.50 (m, 1H), 1.43 (dd, J = 13.7, 11.5 Hz, 2H), 1.40 – 1.36 (m, 2H), 1.34 (d, J = 6.3 Hz, 3H), 1.25 (d, J = 6.6 Hz, 3H), 1.22 (d, J = 6.2 Hz, 3H), 1.17 (d, J = 6.2 Hz, 3H), 1.15 (d, J = 6.6 Hz, 3H), 1.12 (d, J = 6.9 Hz, 3H), 1.00 (d, J = 6.4 Hz, 3H);

13C NMR (150 MHz, CD_3OD) δ 216.24, 167.47, 153.57, 121.98, 104.21, 102.12, 86.87, 82.83, 81.41, 76.35, 74.64, 71.73, 71.06, 70.71, 69.52, 68.26, 67.68, 62.13, 60.84, 59.39, 59.25, 49.76, 46.85, 42.46, 37.31, 36.26, 34.37, 33.69, 27.76, 21.44, 18.79, 18.60, 18.07, 17.49, 16.71.

Note: spectrum recorded obtained in this study matches that reported by Wang *et al*., (2016), aldgamycin K.

^{1}H NMR and ^{13}C NMR data of aldgamycin G (10)

^{1}H NMR (600 MHz, $CDCl_3$) *δ* 6.63 – 6.58 (m, 1H), 6.55 (d, *J* = 15.7 Hz, 1H), 6.41 (dd, *J* = 15.4, 9.3 Hz, 1H), 5.81 (d, *J* = 15.4 Hz, 1H), 5.34 (dq, *J* = 12.4, 6.2 Hz, 1H), 4.60 (d, *J* = 7.7 Hz, 1H), 4.55 (d, *J* = 7.7 Hz, 1H), 4.36 (q, *J* = 6.5 Hz, 1H), 4.15 (dd, *J* = 10.1, 3.0 Hz, 1H), 3.85 (dq, *J* = 12.1, 6.1 Hz, 1H), 3.74 (t, *J* = 2.9 Hz, 1H), 3.62 (dd, *J* = 10.2, 3.0 Hz, 1H), 3.60 (s, 3H), 3.54 (s, 3H), 3.53 – 3.48 (m, 1H), 3.45 (d, *J* = 7.7 Hz, 1H), 3.36 (d, *J* = 10.3 Hz, 1H), 3.27 – 3.23 (m, 1H), 3.18 (d, *J* = 7.6 Hz, 1H), 3.08 – 3.03 (m, 2H), 2.68 (td, *J* = 10.4, 6.6 Hz, 1H), 2.62 – 2.52 (m, 1H), 2.45 (s, 1H), 2.32 (s, 1H), 1.83 (d, *J* =

12.7 Hz, 1H), 1.58 (s, 1H), 1.56 (d, *J* = 6.6 Hz, 3H), 1.53 – 1.49 (m, 1H), 1.46 (dd, *J* = 13.4, 4.0 Hz, 1H), 1.39 – 1.33 (m, 1H), 1.32 (d, *J* = 6.3 Hz, 3H), 1.24 (d, *J* = 6.2 Hz, 4H), 1.20 (d, *J* = 6.2 Hz, 3H), 1.14 (dd, *J* = 13.7, 6.7 Hz, 4H), 0.94 (d, *J* = 6.8 Hz, 3H);<br>^{13}C NMR (150 MHz, $CDCl_3$) δ 200.75, 165.33, 154.02, 150.57, 144.30, 125.54, 120.89, 101.16, 101.04, 86.62, 84.84, 82.01, 81.40, 79.73, 72.79, 71.79, 70.83, 68.85, 67.22, 67.14, 61.82, 59.78, 59.12, 59.02, 49.58, 44.69, 41.65, 41.24, 34.05, 32.05, 20.63, 19.18, 18.51, 17.89, 17.73, 17.04, 13.71.<br>Note: spectrum recorded obtained in this study matches that reported by Mizobuchi et al. (1986) and Zitounni et al. (2007), aldgamycin G.
**^{1}H NMR and ^{13}C NMR data of swalpamycin B (11)**
^{1}H NMR (600 MHz, CD_3OD) δ 7.06 (dd, *J* = 15.0, 11.1 Hz, 1H), 6.60 (dd, *J* = 15.5, 9.9 Hz, 1H), 6.44 (d, *J* = 15.0 Hz, 1H), 6.26 (dd, *J* = 15.3, 11.0 Hz, 1H), 6.06 (dd, *J* = 15.3, 9.4 Hz, 1H), 5.86 (d, *J* = 15.5 Hz, 1H), 5.10 (dq, *J* = 12.7, 6.3 Hz, 1H), 4.59 (d, *J* = 8.0 Hz, 1H), 4.56 (d, *J* = 7.8 Hz, 1H), 3.99 (dd, *J* = 9.8, 3.3 Hz, 1H), 3.90 – 3.82 (m, 2H), 3.76 (t, *J* = 2.7 Hz, 1H), 3.68 – 3.62 (m, 2H), 3.58 (s, 1H), 3.57 (s, 3H), 3.56 – 3.52 (m, 2H), 3.51 (s, 3H), 3.34 (d, *J* = 10.2 Hz, 1H), 3.17 (dd, *J* = 9.6, 2.8 Hz, 1H), 3.06 (dd, *J* = 8.0, 2.8 Hz, 1H), 2.80 – 2.72 (m, 1H), 2.58 – 2.48 (m, 1H), 2.48 – 2.41 (m, 1H), 1.73 – 1.64 (m, 1H), 1.61 – 1.56 (m, 1H), 1.56 – 1.53 (m, 1H), 1.52 – 1.50 (m, 1H), 1.46 – 1.40 (m, 2H), 1.37 (d, *J* = 6.3 Hz, 3H), 1.34 (d, *J* = 6.2 Hz, 1H), 1.25 – 1.19 (m, 7H), 1.19 – 1.13 (m, 8H), 0.99 (d, *J* = 6.8 Hz, 3H);<br>^{13}C NMR (150 MHz, CD_3OD) δ 206.61, 167.65, 153.44, 143.44, 143.04, 134.42, 124.95, 122.39, 103.90, 102.30, 88.31, 82.84, 81.61, 76.36, 74.62, 71.81, 71.05, 70.72, 69.54, 69.50, 67.62, 62.16, 59.58, 52.52, 46.26, 42.32, 37.34, 35.54, 33.72, 21.43, 20.07, 18.64, 18.07, 17.92, 16.72.<br>Note: spectrum recorded obtained in this study matches that reported by Zitounni et al. (2007) and Wang et al., (2013), swalpamycin B.

Reference:

Mizobuchi, S., Mochizuki, J., Soga, H., Tanba, H., and Inoue, H. (1986). Aldgamycin G, a new macrolide antibiotic. *J. Antibiot. (Tokyo)* 39, 1776-1778. doi:10.7164/antibiotics.39.1776

Wang, C.X., Ding, R., Jiang, S.T., Tang, J.S., Hu, D., and Chen, G.D., et al. (2016). Aldgamycins J-O, 16-membered macrolides with a branched octose unit from *Streptomycetes* sp. and their antibacterial activities. *J. Nat. Prod.* 79, 2446-2454. doi:10.1021/acs.jnatprod.6b00200

Wang, X., Tabudravu, J., Jaspars, M., and Deng, H. (2013). Tianchimycins A–B, 16-membered macrolides from the rare actinomycete *Saccharothrix xinjiangensis*. *Tetrahedron* 69, 6060-6064. doi: 10.1016/j.tet.2013.05.094

Zitouni, A., Sabaou, N., Mathieu, F., and Lebrihi, A. (2007). Novel *saccharothrix* strain and antibiotics derived therefrom, i.e. Mutactimycins and aldgamycins. U.S. Patent No 20070202574A1. Washington, DC: U.S. Patent and Trademark Office.

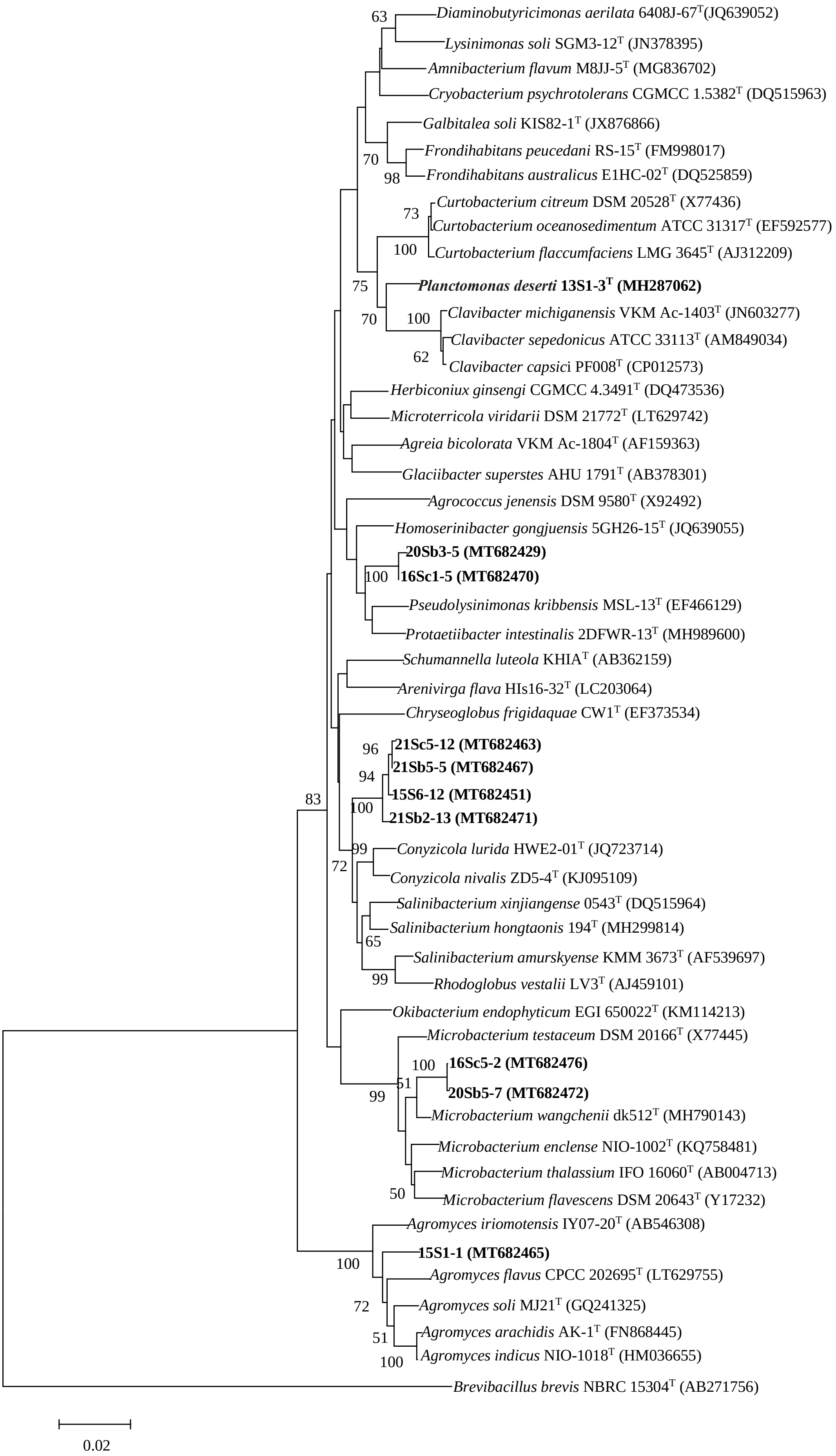

Diaminobutyricimonas aerilata 6408J-67T(JQ639052)
Lysinimonas soli SGM3-12T (JN378395)
Amnibacterium flavum M8JJ-5T (MG836702)
Cryobacterium psychrotolerans CGMCC 1.5382T (DQ515963)
Galbitalea soli KIS82-1T (JX876866)
Frondihabitans peucedani RS-15T (FM998017)
Frondihabitans australicus E1HC-02T (DQ525859)
Curtobacterium citreum DSM 20528T (X77436)
Curtobacterium oceanosedimentum ATCC 31317T (EF592577)
Curtobacterium flaccumfaciens LMG 3645T (AJ312209)
Planctomonas deserti 13S1-3T (MH287062)
Clavibacter michiganensis VKM Ac-1403T (JN603277)
Clavibacter sepedonicus ATCC 33113T (AM849034)
Clavibacter capsici PF008T (CP012573)
Herbiconiux ginsengi CGMCC 4.3491T (DQ473536)
Microterricola viridarii DSM 21772T (LT629742)
Agreia bicolorata VKM Ac-1804T (AF159363)
Glaciibacter superstes AHU 1791T (AB378301)
Agrococcus jenensis DSM 9580T (X92492)
Homoserinibacter gongjuensis 5GH26-15T (JQ639055)
20Sb3-5 (MT682429)
16Sc1-5 (MT682470)
Pseudolysinimonas kribbensis MSL-13T (EF466129)
Protaetiibacter intestinalis 2DFWR-13T (MH989600)
Schumannella luteola KHIAT (AB362159)
Arenivirga flava HIs16-32T (LC203064)
Chryseoglobus frigidaquae CW1T (EF373534)
21Sc5-12 (MT682463)
21Sb5-5 (MT682467)
15S6-12 (MT682451)
21Sb2-13 (MT682471)
Conyzicola lurida HWE2-01T (JQ723714)
Conyzicola nivalis ZD5-4T (KJ095109)
Salinibacterium xinjiangense 0543T (DQ515964)
Salinibacterium hongtaonis 194T (MH299814)
Salinibacterium amurskyense KMM 3673T (AF539697)
Rhodoglobus vestalii LV3T (AJ459101)
Okibacterium endophyticum EGI 650022T (KM114213)
Microbacterium testaceum DSM 20166T (X77445)
16Sc5-2 (MT682476)
20Sb5-7 (MT682472)
Microbacterium wangchenii dk512T (MH790143)
Microbacterium enclense NIO-1002T (KQ758481)
Microbacterium thalassium IFO 16060T (AB004713)
Microbacterium flavescens DSM 20643T (Y17232)
Agromyces iriomotensis IY07-20T (AB546308)
15S1-1 (MT682465)
Agromyces flavus CPCC 202695T (LT629755)
Agromyces soli MJ21T (GQ241325)
Agromyces arachidis AK-1T (FN868445)
Agromyces indicus NIO-1018T (HM036655)
Brevibacillus brevis NBRC 15304T (AB271756)
0.02

Figure S1. Neighbor-joining phylogenetic tree based on 16S rRNA gene sequences of nine potential novel strains and one verified new species of new genus in the family *Microbacteriaceae* isolated from Taklamakan desert soil. Numbers at nodes indicate the level of bootstrap support (> 50%) based on 1000 replications. Bar, 2 nt substitutions per 100 nt.

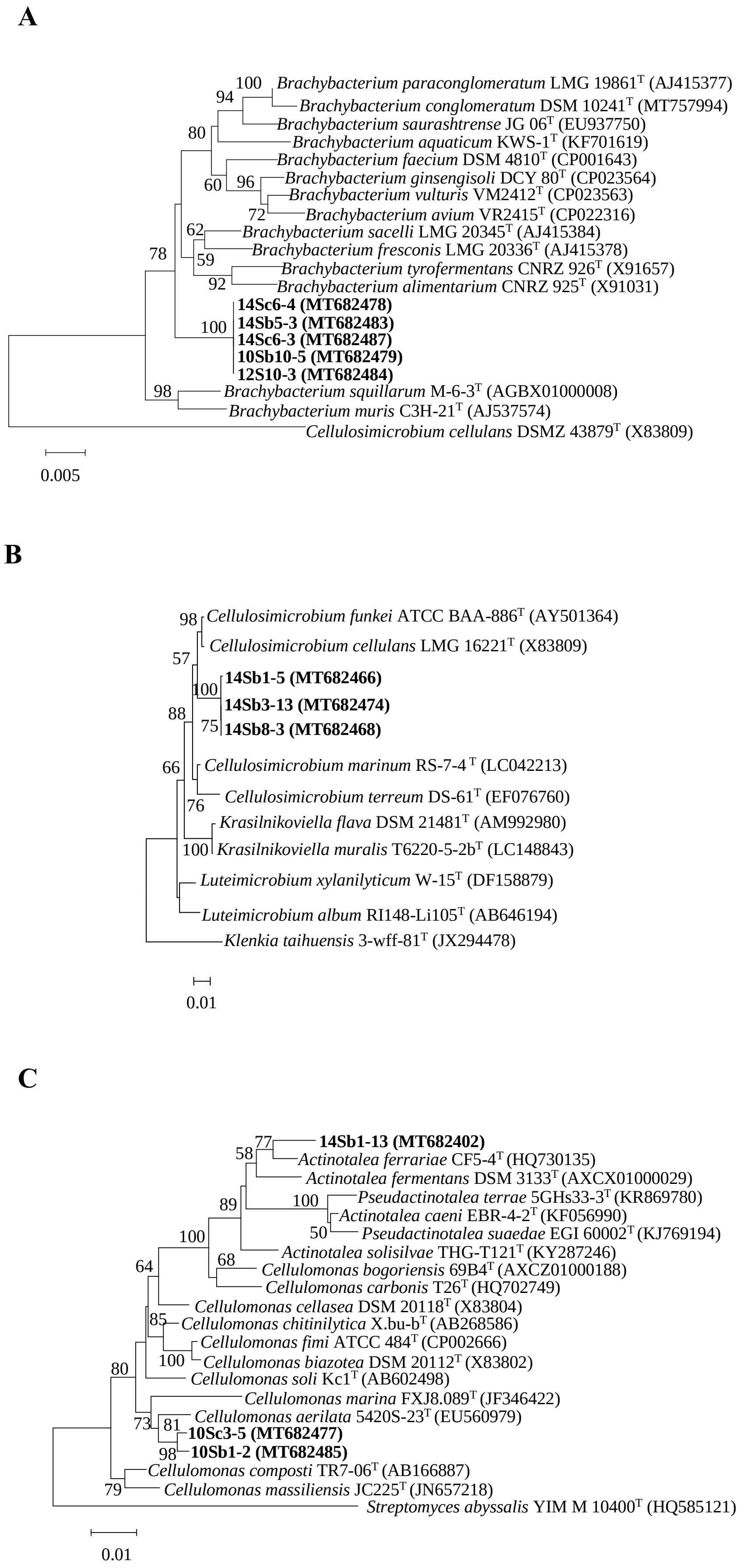
A
Brachybacterium paraconglomeratum LMG 19861T (AJ415377)
Brachybacterium conglomeratum DSM 10241T (MT757994)
Brachybacterium saurashtrense JG 06T (EU937750)
Brachybacterium aquaticum KWS-1T (KF701619)
Brachybacterium faecium DSM 4810T (CP001643)
Brachybacterium ginsengisoli DCY 80T (CP023564)
Brachybacterium vulturis VM2412T (CP023563)
Brachybacterium avium VR2415T (CP022316)
Brachybacterium sacelli LMG 20345T (AJ415384)
Brachybacterium fresconis LMG 20336T (AJ415378)
Brachybacterium tyrofermentans CNRZ 926T (X91657)
Brachybacterium alimentarium CNRZ 925T (X91031)
14Sc6-4 (MT682478)
14Sb5-3 (MT682483)
14Sc6-3 (MT682487)
10Sb10-5 (MT682479)
12S10-3 (MT682484)
Brachybacterium squillarum M-6-3T (AGBX01000008)
Brachybacterium muris C3H-21T (AJ537574)
Cellulosimicrobium cellulans DSMZ 43879T (X83809)
100
94
80
60
96
72
62
59
92
78
100
98
0.005
B
Cellulosimicrobium funkei ATCC BAA-886T (AY501364)
Cellulosimicrobium cellulans LMG 16221T (X83809)
14Sb1-5 (MT682466)
14Sb3-13 (MT682474)
14Sb8-3 (MT682468)
Cellulosimicrobium marinum RS-7-4T (LC042213)
Cellulosimicrobium terreum DS-61T (EF076760)
Krasilnikoviella flava DSM 21481T (AM992980)
Krasilnikoviella muralis T6220-5-2bT (LC148843)
Luteimicrobium xylanilyticum W-15T (DF158879)
Luteimicrobium album RI148-Li105T (AB646194)
Klenkia taihuensis 3-wff-81T (JX294478)
98
57
100
88
75
66
76
100
0.01
C
14Sb1-13 (MT682402)
Actinotalea ferrariae CF5-4T (HQ730135)
Actinotalea fermentans DSM 3133T (AXCX01000029)
Pseudactinotalea terrae 5GHs33-3T (KR869780)
Actinotalea caeni EBR-4-2T (KF056990)
Pseudactinotalea suaedae EGI 60002T (KJ769194)
Actinotalea solisilvae THG-T121T (KY287246)
Cellulomonas bogoriensis 69B4T (AXCZ01000188)
Cellulomonas carbonis T26T (HQ702749)
Cellulomonas cellasea DSM 20118T (X83804)
Cellulomonas chitinilytica X.bu-bT (AB268586)
Cellulomonas fimi ATCC 484T (CP002666)
Cellulomonas biazotea DSM 20112T (X83802)
Cellulomonas soli Kc1T (AB602498)
Cellulomonas marina FXJ8.089T (JF346422)
Cellulomonas aerilata 5420S-23T (EU560979)
10Sc3-5 (MT682477)
10Sb1-2 (MT682485)
Cellulomonas composti TR7-06T (AB166887)
Cellulomonas massiliensis JC225T (JN657218)
Streptomyces abyssalis YIM M 10400T (HQ585121)
77
58
89
100
50
100
68
64
85
100
80
73
81
98
79
0.01

D

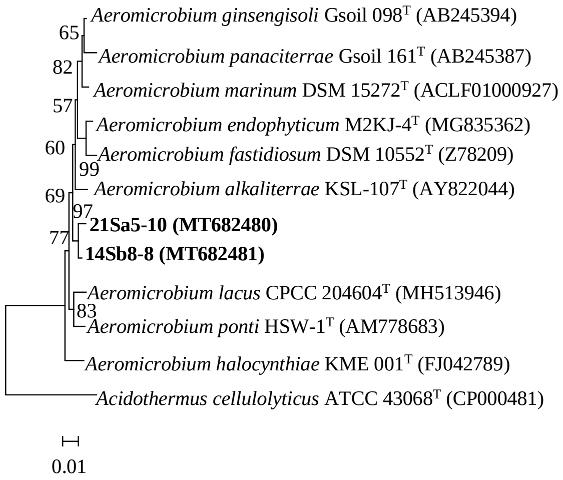
Aeromicrobium ginsengisoli Gsoil 098T (AB245394)
65
Aeromicrobium panaciterrae Gsoil 161T (AB245387)
82
Aeromicrobium marinum DSM 15272T (ACLF01000927)
57
Aeromicrobium endophyticum M2KJ-4T (MG835362)
60
Aeromicrobium fastidiosum DSM 10552T (Z78209)
99
Aeromicrobium alkaliterrae KSL-107T (AY822044)
69
97
21Sa5-10 (MT682480)
77
14Sb8-8 (MT682481)
Aeromicrobium lacus CPCC 204604T (MH513946)
83
Aeromicrobium ponti HSW-1T (AM778683)
Aeromicrobium halocynthiae KME 001T (FJ042789)
Acidothermus cellulolyticus ATCC 43068T (CP000481)
0.01

E

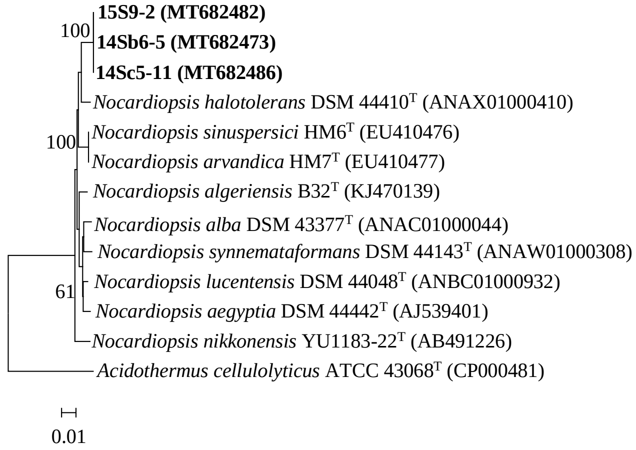
15S9-2 (MT682482)
100
14Sb6-5 (MT682473)
14Sc5-11 (MT682486)
Nocardiopsis halotolerans DSM 44410T (ANAX01000410)
Nocardiopsis sinuspersici HM6T (EU410476)
100
Nocardiopsis arvandica HM7T (EU410477)
Nocardiopsis algeriensis B32T (KJ470139)
Nocardiopsis alba DSM 43377T (ANAC01000044)
Nocardiopsis synnemataformans DSM 44143T (ANAW01000308)
Nocardiopsis lucentensis DSM 44048T (ANBC01000932)
61
Nocardiopsis aegyptia DSM 44442T (AJ539401)
Nocardiopsis nikkonensis YU1183-22T (AB491226)
Acidothermus cellulolyticus ATCC 43068T (CP000481)
0.01

F

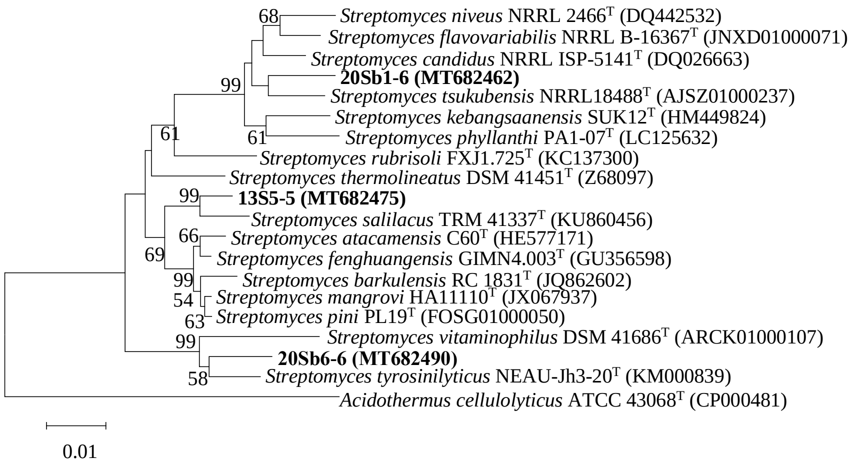
68
Streptomyces niveus NRRL 2466T (DQ442532)
Streptomyces flavovariabilis NRRL B-16367T (JNXD01000071)
Streptomyces candidus NRRL ISP-5141T (DQ026663)
20Sb1-6 (MT682462)
99
Streptomyces tsukubensis NRRL18488T (AJSZ01000237)
Streptomyces kebangsaanensis SUK12T (HM449824)
61
61
Streptomyces phyllanthi PA1-07T (LC125632)
Streptomyces rubrisoli FXJ1.725T (KC137300)
Streptomyces thermolineatus DSM 41451T (Z68097)
99
13S5-5 (MT682475)
Streptomyces salilacus TRM 41337T (KU860456)
66
Streptomyces atacamensis C60T (HE577171)
69
Streptomyces fenghuangensis GIMN4.003T (GU356598)
99
Streptomyces barkulensis RC 1831T (JQ862602)
54
Streptomyces mangrovi HA11110T (JX067937)
63
Streptomyces pini PL19T (FOSG01000050)
99
Streptomyces vitaminophilus DSM 41686T (ARCK01000107)
20Sb6-6 (MT682490)
58
Streptomyces tyrosinilyticus NEAU-Jh3-20T (KM000839)
Acidothermus cellulolyticus ATCC 43068T (CP000481)
0.01

G

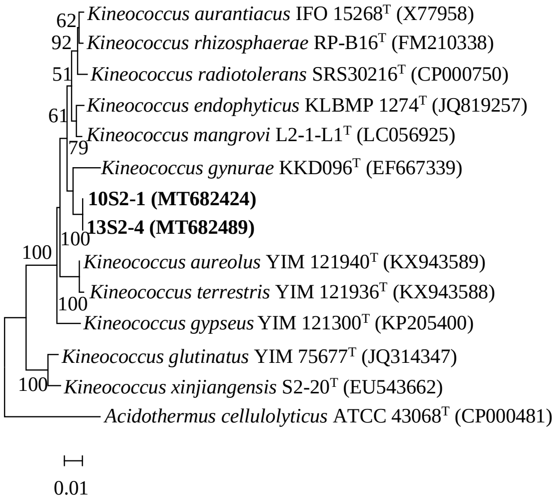
Kineococcus aurantiacus IFO 15268^{T} (X77958)
Kineococcus rhizosphaerae RP-B16^{T} (FM210338)
Kineococcus radiotolerans SRS30216^{T} (CP000750)
Kineococcus endophyticus KLBMP 1274^{T} (JQ819257)
Kineococcus mangrovi L2-1-L1^{T} (LC056925)
Kineococcus gynurae KKD096^{T} (EF667339)
10S2-1 (MT682424)
13S2-4 (MT682489)
Kineococcus aureolus YIM 121940^{T} (KX943589)
Kineococcus terrestris YIM 121936^{T} (KX943588)
Kineococcus gypseus YIM 121300^{T} (KP205400)
Kineococcus glutinatus YIM 75677^{T} (JQ314347)
Kineococcus xinjiangensis S2-20^{T} (EU543662)
Acidothermus cellulolyticus ATCC 43068^{T} (CP000481)
62
92
51
61
79
100
100
100
100
0.01

H

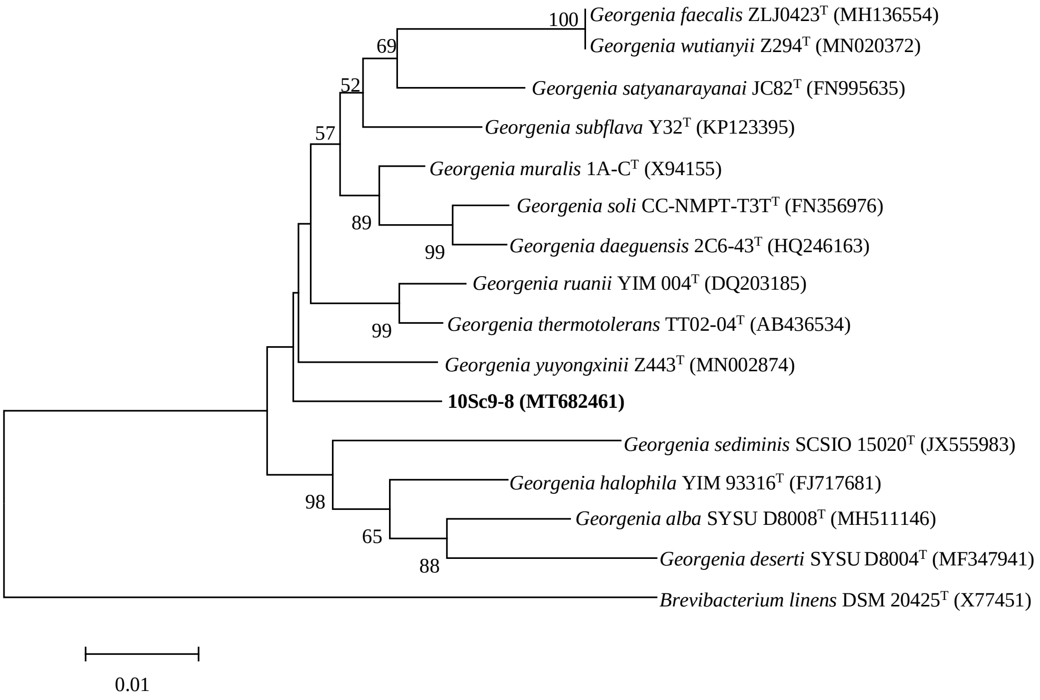
Georgenia faecalis ZLJ0423^{T} (MH136554)
Georgenia wutianyii Z294^{T} (MN020372)
Georgenia satyanarayanai JC82^{T} (FN995635)
Georgenia subflava Y32^{T} (KP123395)
Georgenia muralis 1A-C^{T} (X94155)
Georgenia soli CC-NMPT-T3T^{T} (FN356976)
Georgenia daeguensis 2C6-43^{T} (HQ246163)
Georgenia ruanii YIM 004^{T} (DQ203185)
Georgenia thermotolerans TT02-04^{T} (AB436534)
Georgenia yuyongxinii Z443^{T} (MN002874)
10Sc9-8 (MT682461)
Georgenia sediminis SCSIO 15020^{T} (JX555983)
Georgenia halophila YIM 93316^{T} (FJ717681)
Georgenia alba SYSU D8008^{T} (MH511146)
Georgenia deserti SYSU D8004^{T} (MF347941)
Brevibacterium linens DSM 20425^{T} (X77451)
100
69
52
57
89
99
99
98
65
88
0.01

I

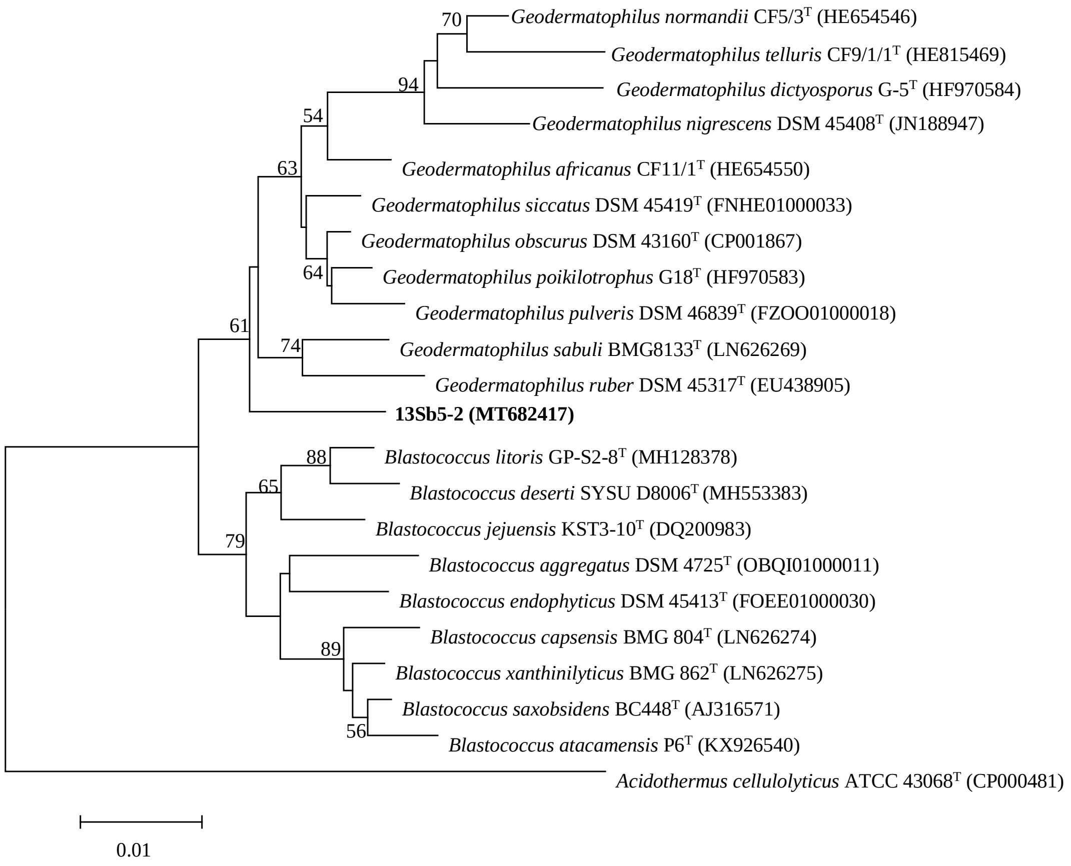

J

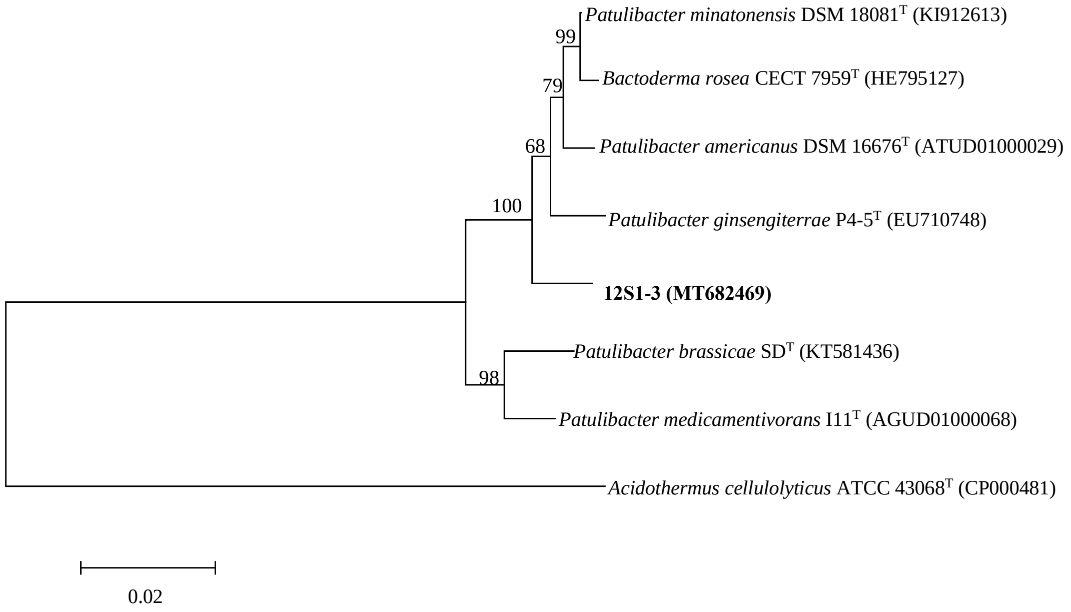

Figure S2. Neighbor-joining phylogenetic tree based on 16S rRNA gene sequences of the other 24 potential novel strains isolated from Taklamakan desert soil. Numbers at nodes indicate the level of bootstrap support (> 50%) based on 1000 replications. **(A)**

Potential novel strains in genus *Brachybacterium* of the family *Dermabacteraceae*; **(B)** Potential novel strains in genus *Cellulosimicrobium* of the family *Promicromonosporaceae*; **(C)** Potential novel strains in genera *Actinotalea* and *Cellulomonas* of the family *Cellulomonadaceae*; **(D)** Potential novel strains in genus *Aeromicrobium* of the family *Nocardioidaceae*; **(E)** Potential novel strains in genus *Nocardiopsis* of the family *Nocardioidaceae*; **(F)** Potential novel strains in genus *Streptomyces* of the family *Streptomycetaceae*; **(G)** Potential novel strains in genus *Kineococcus* of the family *Kineosporiaceae*; **(H)** Potential novel strains in genus *Georgenia* of the family *Bogoriellaceae*; **(I)** Potential novel strains in the family *Geodermatophilaceae*; **(J)** Potential novel strains in genus *Patulibacter* of the family *Patulibacteraceae.*

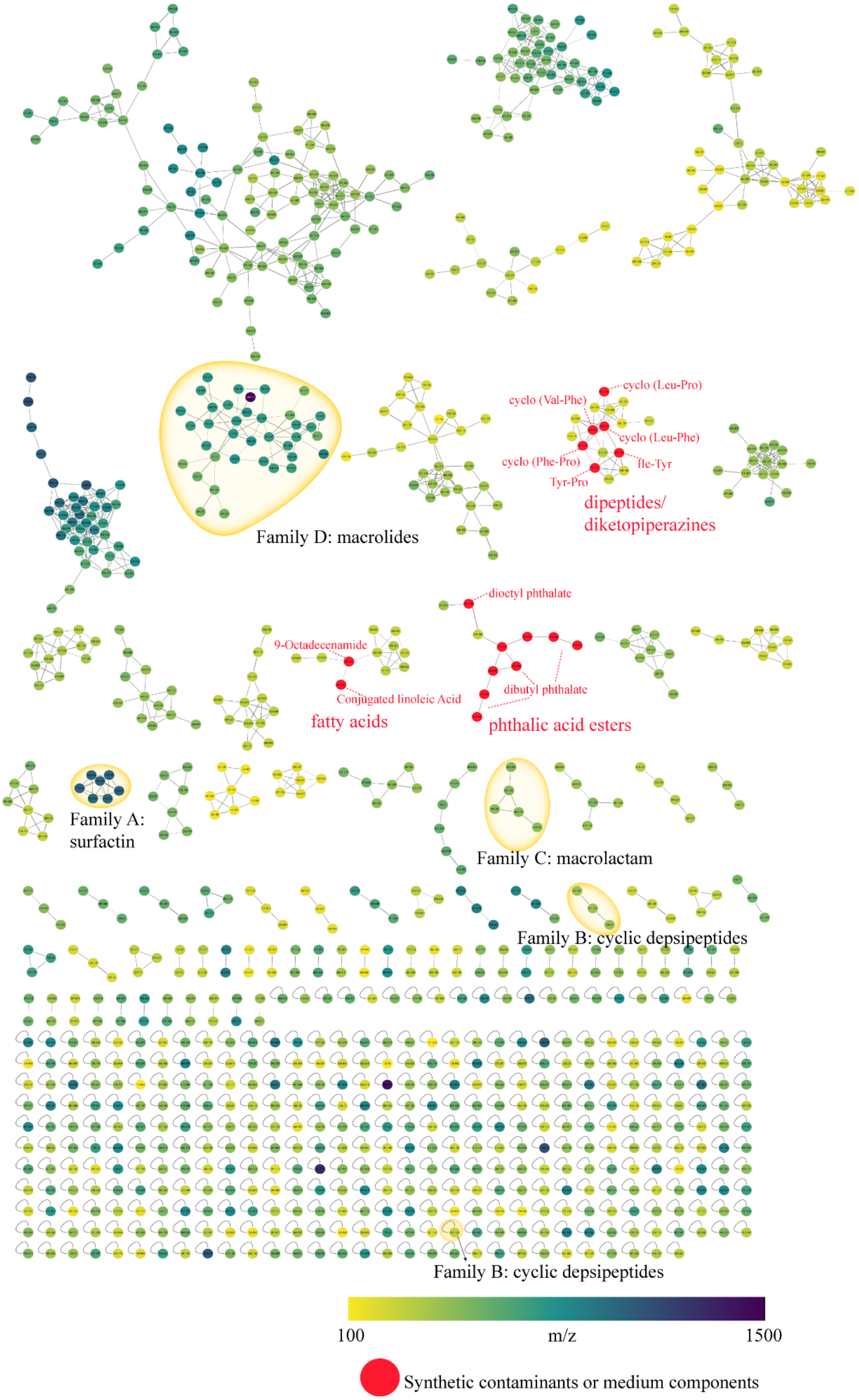

Figure S3. Molecular network of extracts produced by *Saccharothrix* strain 16Sb2-4.

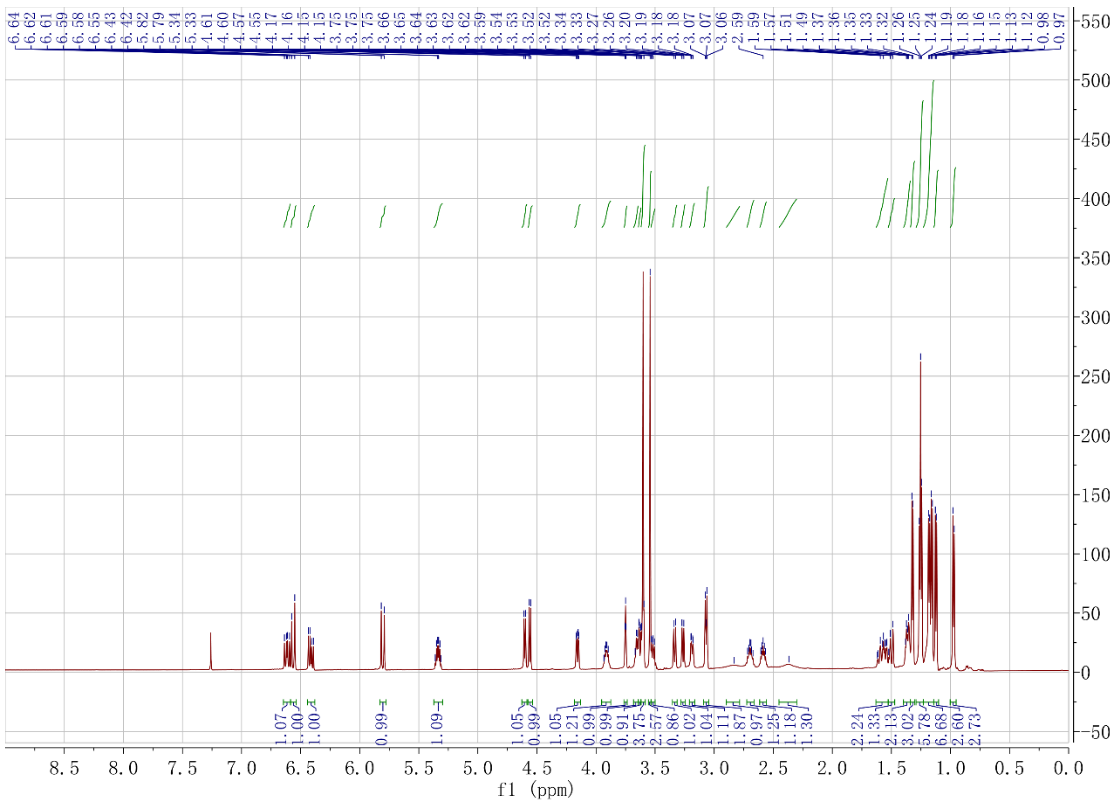

Figure S4. The ^{1}H NMR spectrum of compound **8** in $CDCl_3$ (600 MHz).

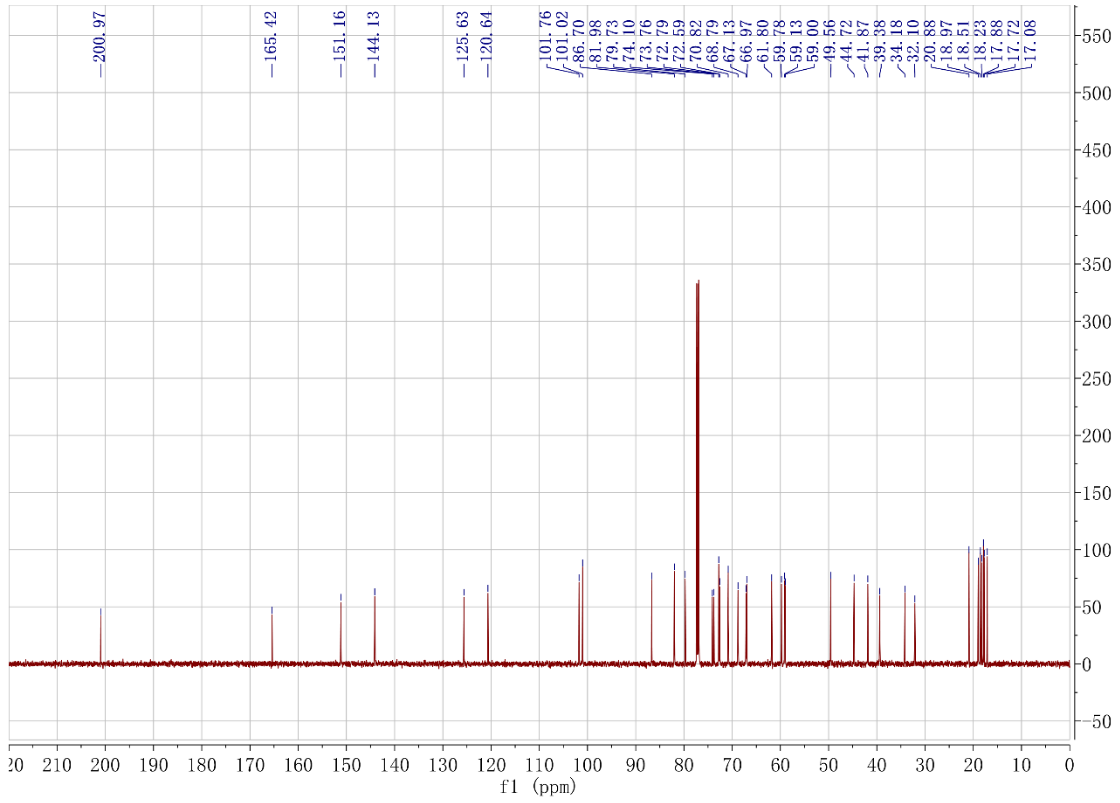

Figure S5. The ^{13}C NMR spectrum of compound **8** in $CDCl_3$ (150 MHz).

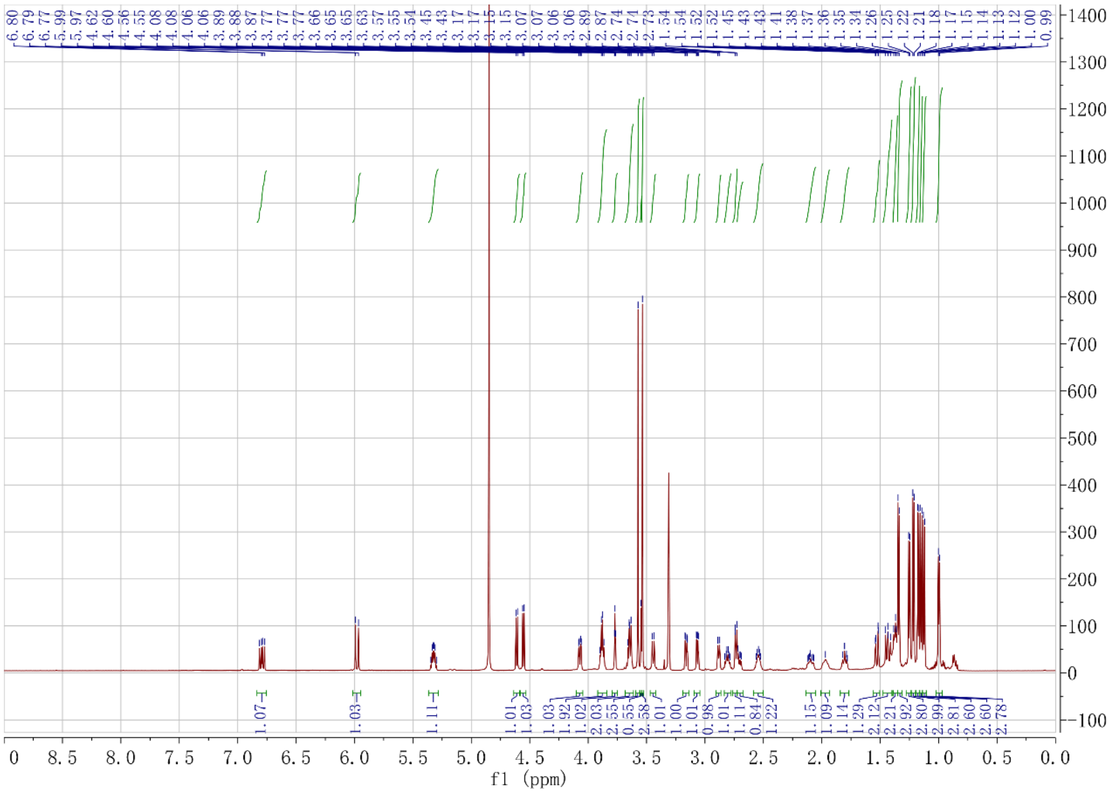

Figure S6. The ^{1}H NMR spectrum of compound **9** in CD_3OD (600 MHz).

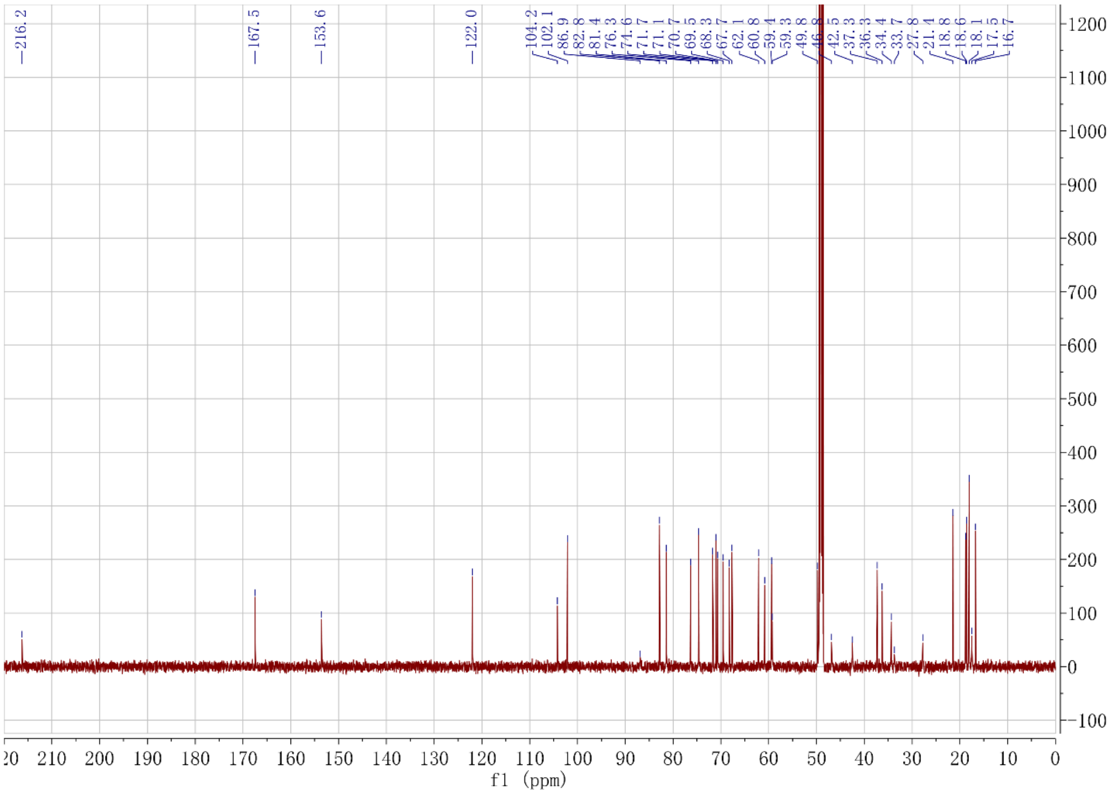

Figure S7. The ^{13}C NMR spectrum of compound **9** in CD_3OD (150 MHz).

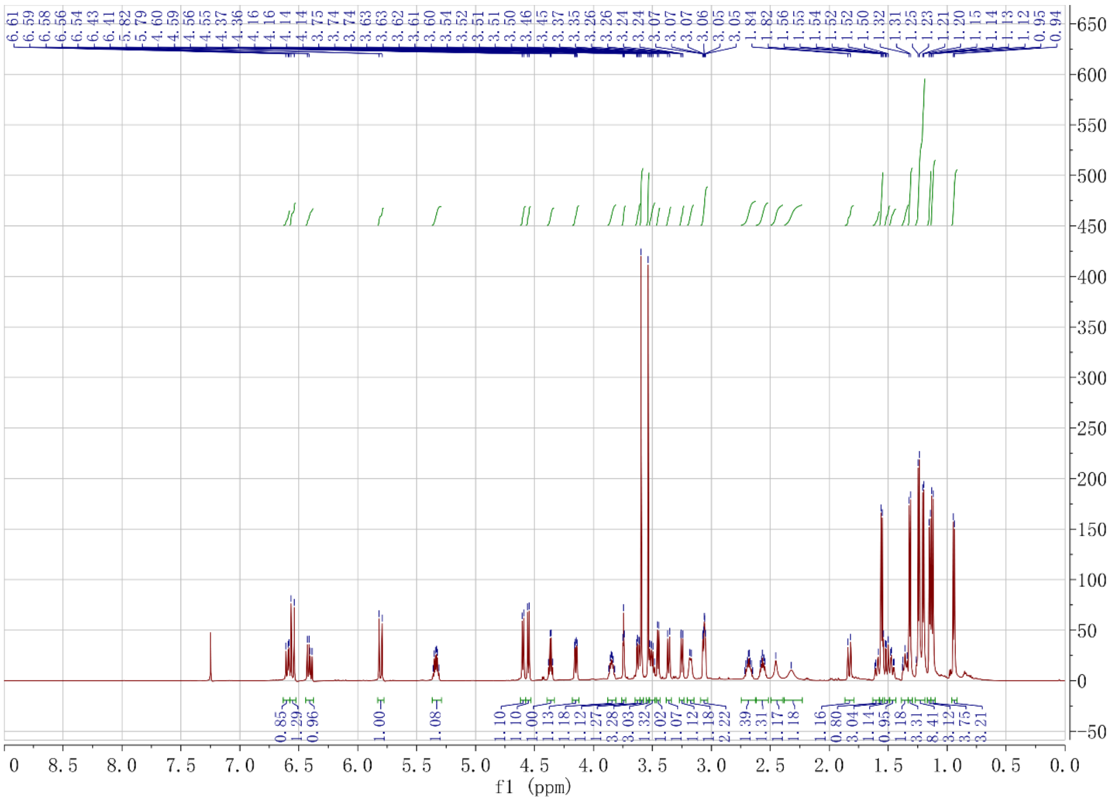

Figure S8. The ^{1}H NMR spectrum of compound **10** in $CDCl_3$ (600 MHz).

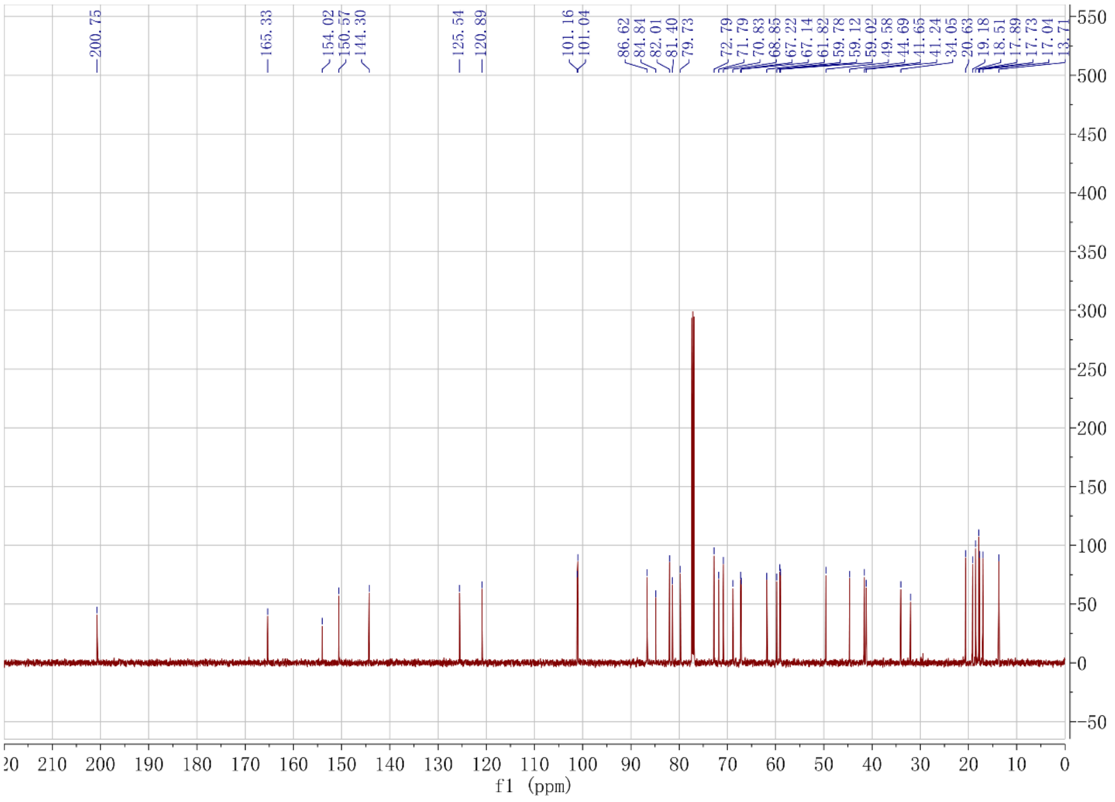

Figure S9. The ^{13}C NMR spectrum of compound **10** in $CDCl_3$ (150 MHz).

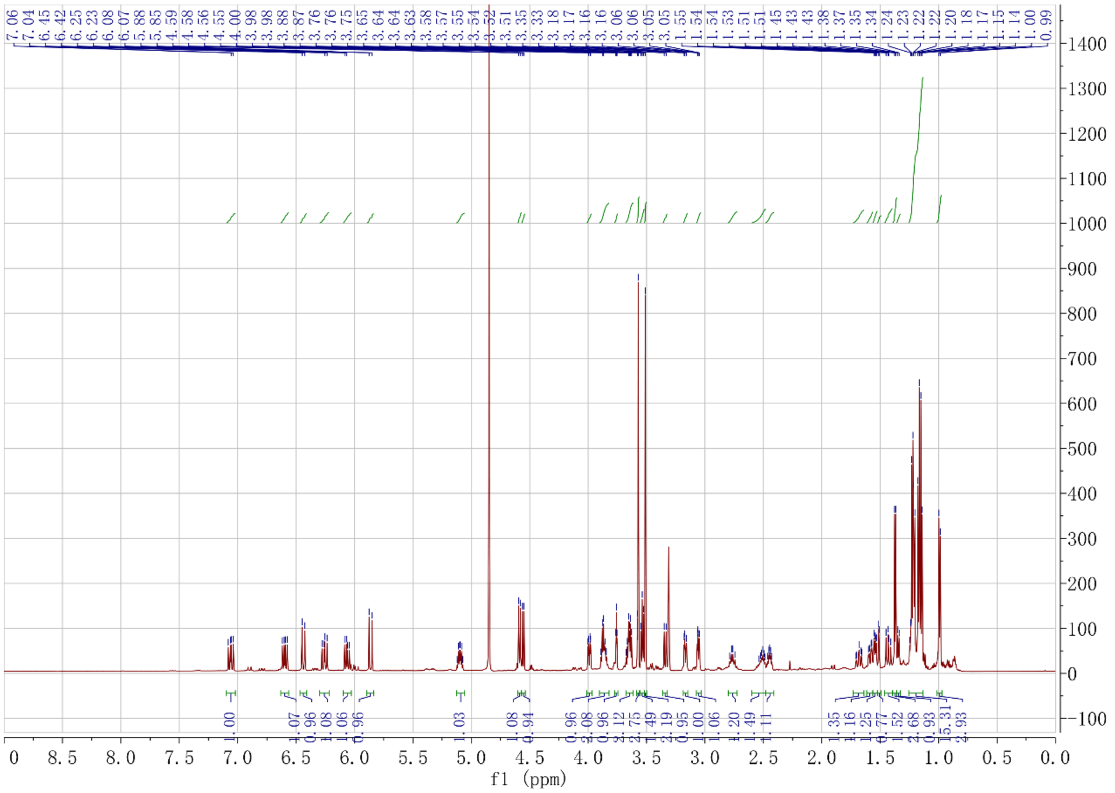

Figure S10. The ^{1}H NMR spectrum of compound **11** in CD_3OD (600 MHz).

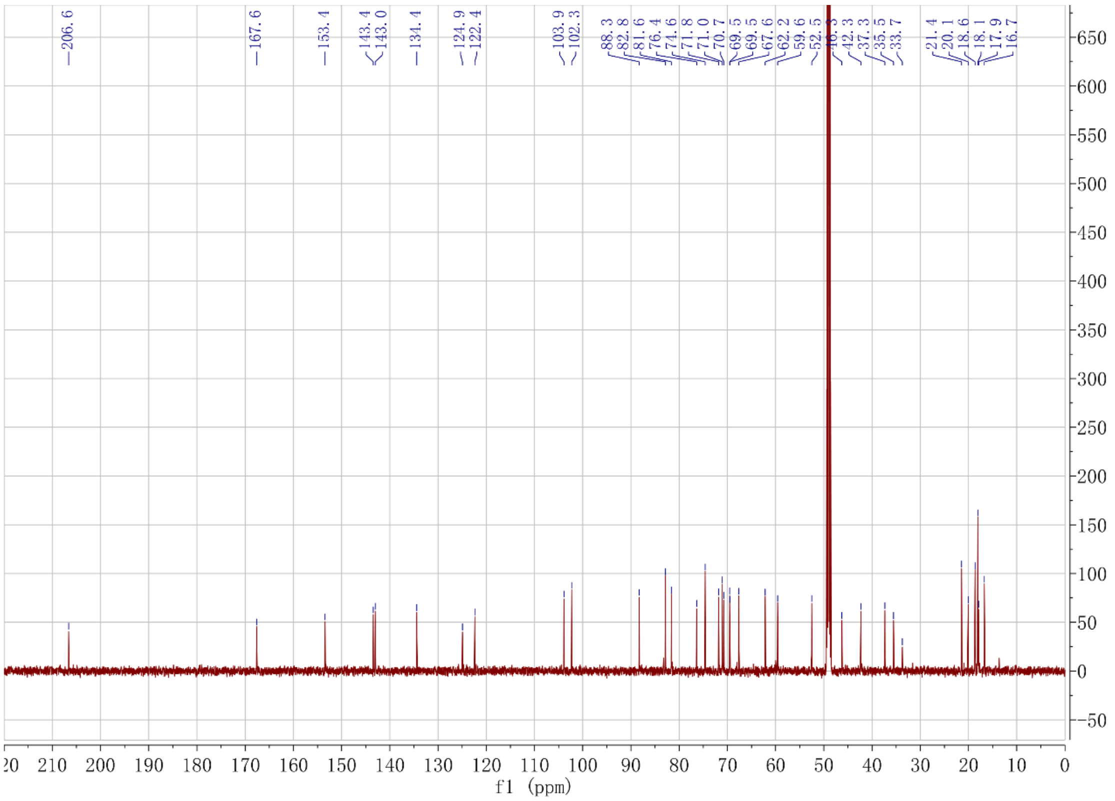

Figure S11. The ^{13}C NMR spectrum of compound **11** in CD_3OD (150 MHz).
